# Structure-mediated modulation of mRNA abundance by A-to-I editing

**DOI:** 10.1038/s41467-017-01459-7

**Published:** 2017-11-02

**Authors:** Anneke Brümmer, Yun Yang, Tracey W. Chan, Xinshu Xiao

**Affiliations:** Department of Integrative Biology and Physiology, Bioinformatics Interdepartmental Program, Molecular Biology Institute, University of California Los Angeles, Los Angeles, CA 90095-1570 USA

## Abstract

RNA editing introduces single nucleotide changes to RNA, thus potentially diversifying gene expression. Recent studies have reported significant changes in RNA editing profiles in disease and development. The functional consequences of these widespread alterations remain elusive because of the unknown function of most RNA editing sites. Here, we carry out a comprehensive analysis of A-to-I editomes in human populations. Surprisingly, we observe highly similar editing profiles across populations despite striking differences in the expression levels of *ADAR* genes. Striving to explain this discrepancy, we uncover a functional mechanism of A-to-I editing in regulating mRNA abundance. We show that A-to-I editing stabilizes RNA secondary structures and reduces the accessibility of AGO2-miRNA to target sites in mRNAs. The editing-dependent stabilization of mRNAs in turn alters the observed editing levels in the stable RNA repertoire. Our study provides valuable insights into the functional impact of RNA editing in human cells.

## Abstract (web summary)

RNA A-to-I editing introduces single nucleotide changes to RNA, but its role in cells remains unclear. Here, the authors analyse A-to-I editomes in human samples and find that A-to-I editing stabilizes RNA secondary structures and reduces the accessibility of AGO2-miRNA to target sites in mRNAs.

## Introduction

RNA editing is a mechanism that alters RNA nucleotides in the co-transcriptional and post-transcriptional stages of gene expression^1,2^. Adenosine-to-inosine (A-to-I) editing is the most abundant type of RNA editing in mammals, catalyzed by the protein family called adenosine deaminases acting on RNA (ADAR). The inosine nucleotide is subsequently recognized as guanine (G) by the translation machinery. Thus, RNA editing can induce amino acid changes in coding regions (referred to as recoding editing sites). A number of such recoding sites have been shown to be essential to cellular function and development^1,3,4^.

Although recoding events clearly show the biological significance of RNA editing, the majority of RNA editing occurs in non-coding regions of the mammalian transcriptome with unknown function^5–7^. Recent genome-wide studies have identified significant global alterations of A-to-I editing levels in various diseases, including cancer, neurological and vascular diseases^8–12^. These discoveries call for detailed investigations of the functional roles of RNA editing, especially those in non-coding regions.

Previous in-depth studies of a small number of editing sites have identified several functional pathways of RNA editing that influence different aspects of gene expression, such as splicing^13–15^, RNA localization^16,17^, and RNA stability^1,12^. In particular, the impact of RNA editing on RNA stability has been the focus of a number of studies, given its potentially profound impact on gene expression. For example, inosine-containing transcripts are digested by the endonuclease V enzyme, providing a direct mechanism for the control of RNA stability by A-to-I editing^18^. A-to-I editing and ADAR proteins can also indirectly affect RNA stability by influencing the abundance or sequences of microRNA (miRNA) molecules, potent regulators of gene expression^1,19–24^.

In addition to affecting miRNA sequences or expression, it has been speculated that A-to-I editing may modify the sequences of miRNA target sites in the 3′ untranslated regions (UTRs) of mRNAs. This hypothesis is attractive as it may explain the functional roles of many editing sites in the non-coding 3′ UTR regions. In fact, specific examples where A-to-I editing creates or destroys miRNA target sites have been found^25–28^. However, there are seemingly contradictory reports on the predicted prevalence of RNA editing sites that alter miRNA target sequences. Some studies proposed that RNA editing tends to avoid miRNA target sites^29,30^, while others suggested that RNA editing is enriched in miRNA target regions^31^. Considering these results, further investigation and rigorous experimental validation are needed to elucidate the functional roles of non-coding editing sites in miRNA targeting.

Recent technologies have enabled the production of an extraordinary amount of RNA sequencing (RNA-Seq) data, which has driven large-scale analyses of RNA editing and its functional mechanisms. One potentially powerful approach is to analyze the differences in RNA editomes present in a large number of individuals. This strategy can uncover the biological, functional or environmental factors that cause variations in RNA editing. In this study, we report a comprehensive analysis of RNA editing across five human populations. Many editing sites are shared among individuals and have similar editing levels, which are not fully explained by the expression of ADAR proteins. Through detailed analyses and corroborating experiments, we characterize a mechanism through which RNA editing in 3′ UTRs affects miRNA targeting and regulates messenger RNA (mRNA) abundance. Instead of introducing nucleotide changes to miRNA target sites, we show that RNA editing stabilizes RNA secondary structures and reduces the accessibility of AGO2-miRNAs to target sites in mRNA 3′ UTRs. This mechanism may explain the functional roles of many RNA editing sites located in non-coding regions. It also highlights the importance of RNA secondary structure in post-transcriptional gene regulation.

## Results

### RNA editing sites differ in prevalence within a population

To obtain a global view of RNA editing in human populations, we analyzed RNA-Seq data sets of 462 human individuals of the 1000 Genomes project. These subjects represent five populations: Utah residents with northern and western European ancestries (CEU; 91 individuals), Finnish in Finland (FIN; 95), British in England and Scotland (GBR; 94), Toscani in Italy (TSI; 93), and Yoruba in Ibadan, Nigeria (YRI; 87). Lymphoblastoid cell lines were established from blood and polyA-selected RNA samples were sequenced^32^. RNA-Seq data were analyzed using our previously developed methods^5,33,34^ (see Methods).

**Fig. 1:**
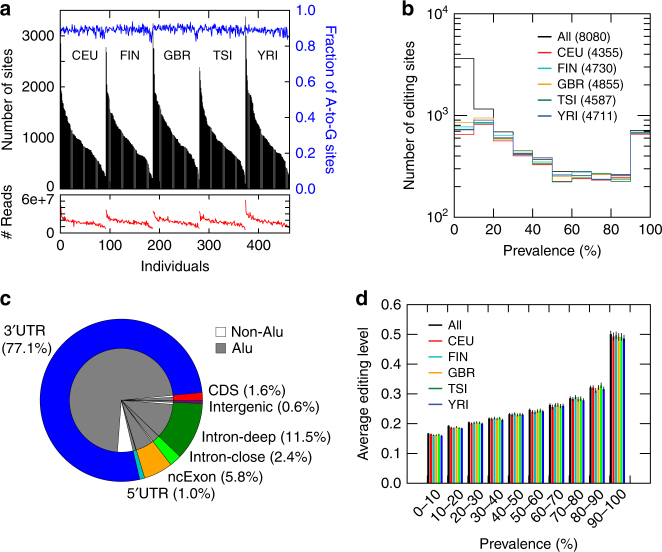
Profile of A-to-I editing in human populations. **a** The total number of RNA-DNA mismatched sites (black bars) identified in RNA-Seq data of each individual in each population, along with the fraction of A-to-G editing sites among all sites (blue), and the number of uniquely mapped reads per sample (red). Individuals are grouped by population first and then sorted by the total number of sites. **a** Prevalence of predicted A-to-I editing sites in each population. Prevalence of an editing site in a population is defined as % of individuals with the site edited among all individuals with read coverage ≥ 10 (i.e., testable sites). The total number of unique A-to-I editing sites in each population or in the union of all individuals is included in brackets. **c** Genomic distribution of predicted A-to-I editing sites. Intron-close: intronic editing sites within 300 nts from exon-intron boundaries; CDS: coding sequence; UTR: untranslated region; Intron-deep: intronic editing sites more than 300 nts away from exon-intron boundaries; ncExon: exons in non-coding transcripts. **d** Average editing level per population for editing sites in different prevalence groups (number of editing sites in each group is shown in **b**). Error bars represent confidence intervals

A total of 28,848 distinct editing sites were identified in the 462 samples. In a single subject, the number of predicted editing sites ranged from ~ 200 to ~ 3500, which approximately correlated with RNA-Seq read coverage of the samples (Fig. 1a). On average, 90% of the editing sites in each individual were of the A-to-G type, reflecting A-to-I editing (Fig. 1a). This high percentage suggests a high accuracy of our RNA editing identification method, as previously shown^34^. Henceforth, we will restrict our analyses to A-to-G sites, since non-A-to-G substitutions constituted a minor fraction and demonstrated little overlap among individuals in general.

To enable inter-individual comparisons, we further focused on editing sites that satisfy the following criteria: (1) ≥ 2 reads with the edited nucleotide and an editing level ≥ 0.1 in ≥ 3 individuals of a population; and (2) read coverage ≥ 10 in ≥ 10% individuals of a population. These cutoffs were chosen to achieve a trade-off between the total number of selected sites and the fraction of A-to-G sites (Supplementary Fig. 1a–c). It should be noted that the %A-to-G sites among all unique sites in a population was lower than that among all sites in one individual (Supplementary Fig. 1b). This is because A-to-G sites, but not non-A-to-G sites, often occurred in multiple individuals. More than 4300 editing sites in each population satisfied the above criteria (Fig. 1b). A total of 8080 editing sites were identified in the union of all populations, 97% and 61% of which overlapped those in the RADAR^35^ and DARNED^36^ databases, respectively. These 8080 editing sites were located in 909 genes (Supplementary Fig. 1d) and most often resided in 3′ UTRs (77%), followed by introns (14%; Fig. 1c). Only a small fraction (1.6%) was found in the coding regions of mRNAs and in 5′ UTRs (1%). Interestingly, among genes with the largest number of editing sites, many have known functions related to immune response with editing sites exclusively in the 3′ UTRs (Supplementary Fig. 1d). It should be noted that the above editing sites are a conservative representation of all editing sites in the populations, due to limited sequencing depth and possible preclusion of sites in hyper-edited regions^37^.

We next analyzed the prevalence of editing sites within each of the five populations. We defined the prevalence of each editing site as the percentage of individuals with the sites edited among those that were testable for this editing event (i.e., ≥10 read coverage). The five populations demonstrated similar distributions of editing site prevalence (Fig. 1b), with the majority of sites having prevalence <50%. However, a considerable subset of editing sites (~ 700) in each population had a prevalence of 90% or higher. In addition, prevalent editing sites showed higher editing levels than relatively rare sites (Fig. 1d). Note that detection of low editing levels depends on read coverage, which may confound the measure of prevalence. As rare sites have relatively low editing levels, edited reads may not be observed in all truly edited individuals given limited read coverage. Thus, the prevalence shown here, especially for rare sites, may be an under-estimate of the true prevalence, although the majority of rare sites defined here should have relatively low true prevalence (Supplementary Fig. 2).

### Prevalent editing sites represent strong ADAR substrates

We next examined the genomic, regulatory and functional features of rare (≤10% prevalence) and prevalent (>90% prevalence) editing sites. The regional location within a transcript was highly similar for the two groups (Supplementary Fig. 3a). In addition, there was no significant difference in expression levels of genes harboring these editing sites (Supplementary Fig. 3b). This observation indicates that the prevalence measure is not confounded by gene expression level. Indeed, rare editing sites had slightly higher average read coverage than prevalent sites (Supplementary Fig. 3c), consistent with our earlier observation that rare editing sites had relatively low editing levels (Fig. 1d) and thus require more reads to be detected.

**Fig. 2:**
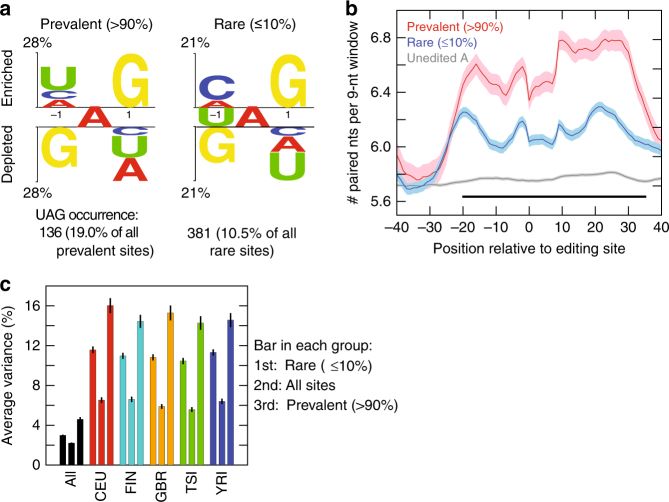
Comparison of prevalent and rare editing sites. **a** Sequence preference for positions flanking prevalent and rare A-to-I editing sites, represented using a two-sample logo program^69^. A total of 714 prevalent (prevalence > 90%) and 3618 rare (prevalence ≤ 10%) editing sites were included, respectively. The number of occurrences of the ‘UAG’ motif is shown below each graph. The ‘UAG’ sequence is significantly enriched around prevalent sites compared to rare sites (*p* = 1.4e-9, Fisher’s Exact test). Prevalent and rare editing sites were defined using editing sites of the union of all individuals (same below). **b** Average number of paired nucleotides in a sliding window of 9 nucleotides (corresponding to the length of ADAR binding region^70^) near prevalent (red) and rare (blue) editing sites, and near random adenosines (gray) located 200–300 nucleotides from editing sites. Shaded areas represent the standard error of the mean. Horizontal black bar indicates the region where there are significant differences between the numbers of paired nucleotides around prevalent and rare editing sites (*p* < 0.01, Wilcoxon Rank-Sum test). **c** Variance in editing levels across individuals explained by *ADAR* expression levels (*ADAR1*, *ADAR2*, and *ADAR3*) in a linear model. Average values of rare (3618 sites), all (8080) and prevalent sites (714) are shown (ordered as the 1st, 2nd, and 3rd bar in each group, respectively). Error bars represent confidence intervals

The sequence contexts (±1 nt) of rare and prevalent editing sites showed a significant difference (Fig. 2a). In particular, prevalent sites were associated with the typical sequence signature known for ADAR substrates^38,39^. The motif UAG was significantly more enriched around prevalent sites than rare sites. Furthermore, compared with rare editing sites, sequences flanking prevalent sites were in stronger double-stranded structures (Fig. 2b). Notably, the sequence neighborhood of both rare and prevalent editing sites showed a higher propensity for double-stranded structures than random adenosines located 200–300 nt away from editing sites (Fig. 2b). This observation indicates that rare editing sites are also ADAR target sites. Altogether, the above sequence and structural properties suggest that prevalent editing sites are stronger ADAR substrates compared to rare sites. It should be noted that the above sequence and structural differences between rare and prevalent editing sites may partially reflect the properties of sites with different editing levels. As prevalent sites have higher editing levels than rare sites (Fig. 1d), it is expected that they are stronger ADAR substrates. Consistently, based on linear regression, *ADAR* expression levels explained a relatively larger fraction of the variance in editing levels of prevalent sites than at rare ones (Fig. 2c). Nevertheless, the stronger correlation between *ADAR* expression and editing levels for prevalent sites may have been partially enabled by the presumably higher accuracy of their estimated editing levels. Overall, *ADAR* expression explained a relatively small fraction of editing variance (on average ~ 11% for all sites in each population). The same observation holds if ADAR protein expression levels were used (Supplementary Fig. 4a), although *ADAR* mRNA and proteins levels show moderate correlation (Supplementary Fig. 4b), or if an editing index^9^ was used instead of the average editing level (Supplementary Fig. 4c). In general, this low contribution of *ADAR* expression to editing variance could be due to low *ADAR* expression variability across individuals or existence of other regulators that mediate editing variation.

Compared to those with rare editing sites, genes harboring prevalent editing sites were enriched in functional categories related to cell-cycle regulation and cell proliferation. Intriguingly, the most significantly enriched categories were sexual reproduction and negative regulation of viral genome replication (Supplementary Fig. 5). No enriched categories were found among genes with rare editing sites. Our findings are in line with recent studies that reported the involvement of *ADAR1* in innate immunity^40–42^ and that A-to-I editing occurs specifically during sexual reproduction in the wheat scab fungus *Fusarium graminearum* ^43^.

### Populations share editing sites with low-level differences

**Fig. 3:**
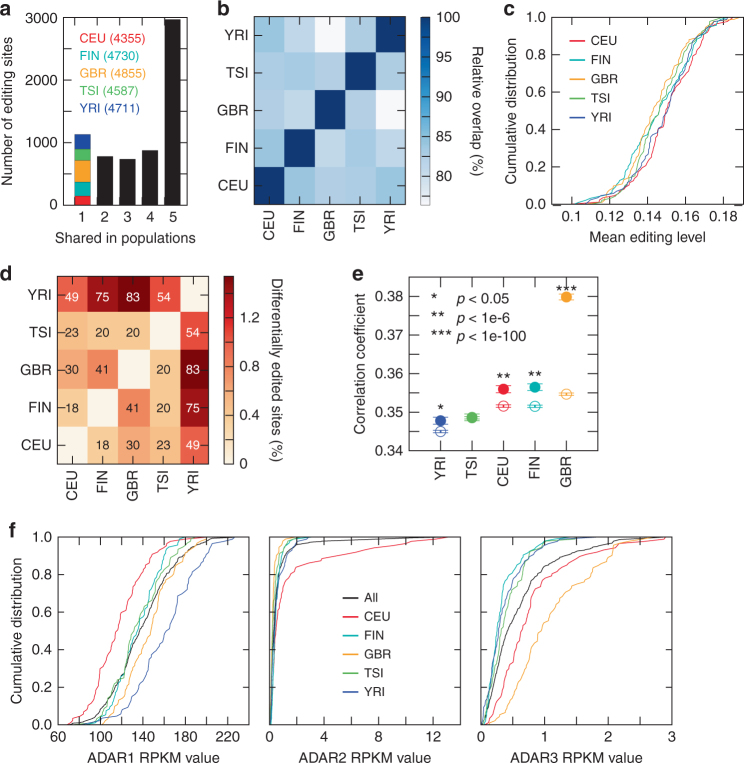
Comparison of editomes across populations. **a** Number of A-to-I editing sites shared between populations. Editing sites specific to one population are color-coded. The total number of editing sites included for each population is shown in parenthesis. **b** Overlap of editing sites between a pair of populations. Percentages calculated relative to the number of editing sites in the population with the smaller total number of sites. The overlap was significant (*p* < 1e-20, Hypergeometric test) for all comparisons. **c** Cumulative distributions of average editing levels (per individual) in different populations. **d** Percentage of differentially edited sites between a pair of populations (*p* < 0.01, Wilcoxon Rank-Sum test, see Methods), relative to the total number of testable sites in each pairwise comparison. The number of differentially edited sites is indicated in each grid. **e** Correlation coefficients of editing levels in two individuals from the same population (filled circles) and of two individuals from different populations (with one individual from the indicated population, the other from any other population, empty circles). Mean and standard error are shown. *P* values are shown to compare the difference between intra- and inter-population correlations (Wilcoxon Rank-Sum test). **f** *ADAR* expression levels in different populations

With the above analyses focused on within-population comparisons, we next examined the differences and similarities of RNA editomes between populations. Among all editing sites included in Fig. 1b, the majority was edited in at least 2 populations, with nearly 3000 sites shared by all five populations (Fig. 3a). Only about 100–300 sites were specific to any one of the five populations. These observed population-specific editing sites were unlikely a consequence of inadequate power for their detection in other populations (Supplementary Fig. 6). A pairwise comparison of RNA editing sites revealed significant overlap between all pairs of populations (Fig. 3b). The individual-specific mean editing levels or editing indexes^9^ were also highly similar across populations (Fig. 3c and Supplementary Fig. 7). A differential test revealed that only a small number of editing sites has significantly different editing levels across populations (Fig. 3d).

Overall, the YRI population showed the highest level of difference in RNA editing relative to other populations (Fig. 3b, d). In particular, YRI and GBR populations appeared to be most different among all pairwise comparisons, with the smallest overlap in editing sites and the largest number of differentially edited sites, and the largest difference in average editing levels (Fig. 3b–d). It should be noted that although the cross-population difference is small, this level of difference is still larger than that between individuals within the same population (Fig. 3e).

### Population-wide editing similarity is not explained by ADARs

**Fig. 4:**
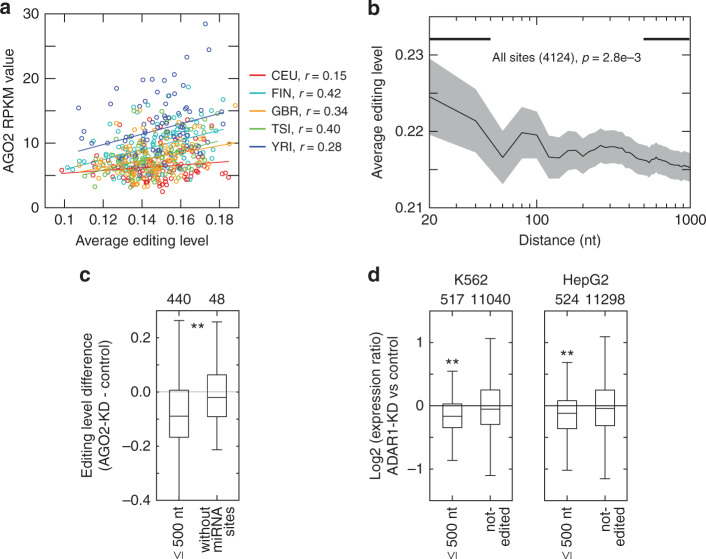
Correlation between AGO2-miRNAs targeting and RNA editing levels. **a** Correlation of average editing levels (per individual) with *AGO2* RPKM levels. Pearson correlation coefficients are shown. **b** Average editing level in dependence of the distance between an editing site and a predicted miRNA target site. The average was taken over all editing sites with distances ≤ the distance shown on the x-axis. Shaded areas represent standard error of the mean. *P* value is shown to compare editing level difference at editing sites close (<50 nt, black bar at top) and relatively far (500–1000 nt, black bar at top) from predicted miRNA target sites (Wilcoxon Rank-Sum test). **c** Editing level differences (*AGO2* KD minus control, K562 cells) of two groups of editing sites identified in the Geuvadis RNA-Seq data. Left: editing sites located in 3′ UTRs within 500 nt from predicted miRNA target sites; right: editing sites in 3′ UTRs without predicted miRNA target sites. The number of genes in each group is shown on top. *P* value was calculated to compare the editing level differences between the two groups of editing sites (Wilcoxon Rank-Sum test). **: *p* < 1e-3. **d** Gene expression changes upon *ADAR1* KD compared to control (K562 and HepG2 cells, respectively) in two groups of genes (with number of genes indicated on top). Group 1 consists of genes with editing sites (identified in the Geuvadis RNA-Seq data) within 500 nt from predicted miRNA target sites (left box in each panel); group 2 consists of genes without editing sites in 3′ UTRs (right box in each panel). Expression ratio was calculated as RPKM ratio of KD to control. *P* values were calculated to compare the log2 fold changes in RPKM values of the two groups of genes (Wilcoxon Rank-Sum test). **: *p* < 1e-7

To understand the population-wise differences in RNA editing, we next examined expression levels of the *ADAR* genes, combining those of *ADAR1* isoforms, p110 and p150. Surprisingly, all three *ADARs* showed significantly different levels across populations (Fig. 3f), which is in stark contrast to the relatively similar average editing levels among the populations (Fig. 3c). Given these observations, we asked whether other RNA binding proteins (RBPs) contribute to the observed editing variability. To this end, we examined expression levels of 507 RBPs using the RNA-Seq data from this study (requiring average RPKM level > 1). A total of 13 RBPs were significantly different in ≥4 pairwise comparisons between populations (Supplementary Table 1). Among these proteins, AGO2 was most significant in the CEU vs. YRI comparison. Notably, between the CEU and YRI populations, we observed the largest difference in *ADAR* expression levels, but the most similar editing levels, compared with other pairs of populations (Fig. 3c). Interestingly, *AGO2* expression was positively correlated with average editing levels (*r* = 0.15–0.42, Fig. 4a), similarly as for *ADAR1* (*r* = 0.18–0.31, Supplementary Fig. 4b).

### Correlation between AGO2-miRNA targeting and editing

We next examined the potential relationship between *AGO2* and RNA editing. As the canonical function of *AGO2* is miRNA-guided transcript destabilization occurring primarily in the cytoplasm, while editing presumably occurs earlier in the life of an mRNA, we hypothesized that the apparent correlation between *AGO2* expression and RNA editing levels reflects RNA editing-dependent AGO2-miRNA targeting, rather than AGO2-dependent RNA editing.

To test this hypothesis, we obtained a list of predicted miRNA target sites (combining TargetScan^44^ and miRCode^45^ predictions) in 3′ UTRs that also harbor RNA editing sites. Interestingly, we observed that editing sites closer to miRNA target sites had higher editing levels than more distant sites (Fig. 4b). This observation also holds if only non-Alu editing sites were analyzed (Supplementary Fig. 8), excluding the possibility that this tendency may have originated from a difference in mappability of reads originating from Alu repeats. Similarly, we analyzed *AGO2* knockdown (KD) and control data sets obtained from K562 cells by the ENCODE project. Consistent with the observed positive correlation between *AGO2* expression and editing levels in the human populations (Fig. 4a), we detected a global reduction in editing levels upon *AGO2* KD in K562 cells for editing sites that are within 500 nt from predicted miRNA target sites (Fig. 4c). This editing difference was not observed at editing sites in genes without predicted miRNA target sites in their 3′ UTRs (Fig. 4c). These AGO2-related editing changes may be explained by an editing-dependent AGO2-miRNA targeting.

To provide complementary evidence for the correlation between editing levels and AGO2-miRNA targeting, we analyzed *ADAR1* KD data obtained from K562 and HepG2 cells by the ENCODE project. We asked whether miRNA target gene expression was altered in case of reduced RNA editing. Figure 4d shows that miRNA target genes that harbor editing sites within 500 nt from miRNA target sites had reduced expression levels upon *ADAR1* KD. As a control, this observation did not hold for genes without editing sites in their 3′ UTRs. The expressions of the above edited miRNA target genes demonstrated consistent directional changes comparing *ADAR1* KD data of K562 and HepG2 cells (Supplementary Fig. 9a).

Altogether, the above data support a model in which AGO2-miRNA preferentially targets the unedited version of the mRNA. This model explains the apparent positive correlation of *AGO2* expression with RNA editing levels and the observed reduction in gene expression levels of miRNA target genes upon *ADAR1* KD.

### AGO2-miRNAs preferentially target unedited mRNA

**Fig. 5:**
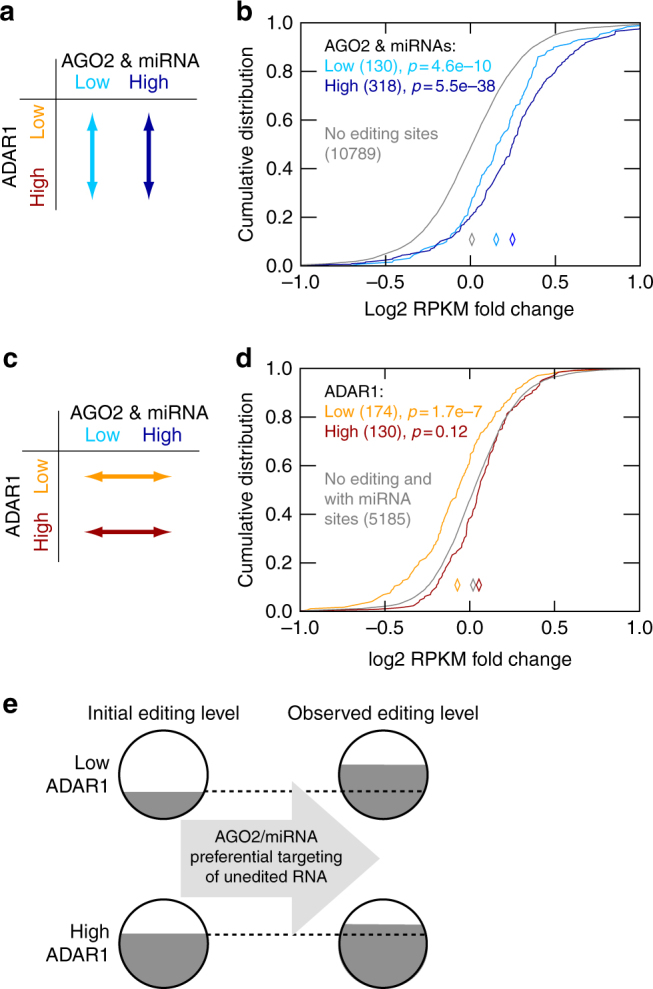
AGO2-miRNA preferentially targets unedited RNA for degradation. **a** Illustration of the comparisons shown in **b**. **b** Comparison of target gene expression levels between individuals with low and high *ADAR1* expression levels. *X*-axis shows log2 fold change of gene expression levels (target genes grouped by *ADAR1* level: high/low). The data were further separated into three categories consisting of individuals with similarly low or high *AGO2* and miRNA expression levels (and requiring miRNA target sites and editing sites to be within 500 nt), and those with no editing sites in these genes. The number of gene-miRNA combinations included in each group is shown in parenthesis. Diamonds represent the medians of the respective curves. *P* values are shown for comparisons against the “no-editing” group (Wilcoxon Rank-Sum test). **c** Illustration of the comparisons shown in **d**. **d** Same as **b**, but for different groups of individuals as illustrated. *X*-axis shows log2 fold change of gene expression levels (target genes grouped by *AGO2* & miRNA levels: high/low). **e** The impact of AGO2-miRNA targeting on observed RNA editing levels according to the following model: AGO2-miRNA preferentially targets unedited version of the transcripts and has more pronounced impact on sites with low initial editing level (upper) than those with high initial editing level (lower). Thus, the AGO2-miRNA targeting buffers the difference in the initial editing levels of the two categories of editing sites

To further support the above model, we carried out a series of analyses using the population-based RNA-Seq data. All analyses included genes harboring editing sites in the 3′ UTRs that are within 500 nt of predicted miRNA target sites. We asked two questions: (1) among individuals with similar *AGO2* and miRNA expression levels, is there a gene expression difference between those with high and low *ADAR1* expression (Fig. 5a, b)? (2) Among individuals with similar *ADAR1* expression levels, is there a gene expression difference between those with high vs. low *AGO2* and miRNA expression levels (Fig. 5c, d)?

For the first question, according to the model where AGO2-miRNAs preferentially target unedited mRNAs, we expect to observe higher target gene expression levels for individuals with higher *ADAR1* expression. The data are consistent with this expectation (Fig. 5b). Furthermore, as expected, the target expression difference was more pronounced in the comparison, where *AGO2* and miRNA levels were high. As a negative control, no gene expression difference depending on *ADAR1* expression levels was observed for genes with no editing sites close to miRNA targets (Fig. 5b). For the second question, we observed significantly lower gene expression levels for individuals with higher (vs. lower) *AGO2* and miRNA levels, as expected for miRNA target genes (Fig. 5d). Importantly, this difference was significant only if *ADAR1* expression was low, consistent with the hypothesis that AGO2-miRNA preferentially destabilizes the unedited version of mRNAs (that are relatively less available for targeting if *ADAR1* is high).

Importantly, data from the population-level analysis (Fig. 5) and those from protein KD experiments (Fig. 4d) showed consistent results. We compared genes with lower expression in individuals with lower (vs. higher) *ADAR1* levels to genes whose expression was reduced upon *ADAR1* KD (vs. control) (Supplementary Fig. 9b, c). The overlap was significantly larger among genes with 3′ UTR editing sites and miRNA target sites within 500 nt than among control genes. The same observation holds for genes with AGO2-dependent expression changes (Supplementary Fig. 9d).

The above observations provide additional support for our earlier hypothesis that AGO2-miRNA preferentially destabilizes unedited mRNAs. Importantly, the impact of this regulation is most evident if *ADAR1* level is low (and, of course, if AGO2-miRNA levels are high). This editing-dependent modulation of mRNA abundance in turn induces an apparent increase in observed editing levels, thereby reducing the observed difference in editing levels of individuals with high vs. low *ADAR1* expression. Figure 5e summarizes the above model and highlights the apparent increase in editing levels due to AGO2-miRNA-mediated destabilization of primarily unedited RNA, especially in the presence of low *ADAR1* levels. Importantly, this model is also consistent with the earlier observation that *AGO2* expression correlates positively with average editing levels (Fig. 4a).

### Structure changes due to editing alter AGO2-miRNA targeting

**Fig. 6:**
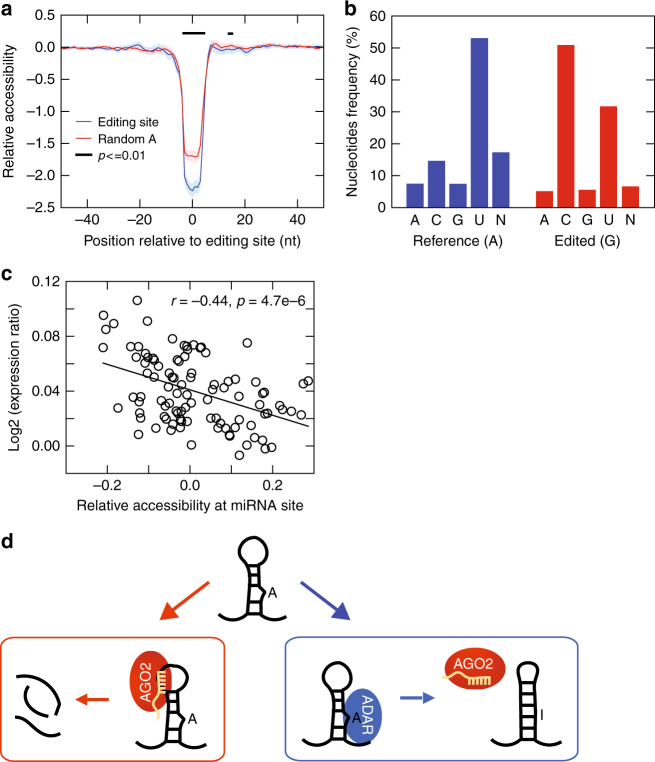
Editing-dependent RNA structural changes near AGO2-miRNA target sites. **a** Profile of relative 7mer accessibility around editing sites located in 3′UTRs and within 100 nt from predicted miRNA target sites (blue), and around random A positions (red). *Y*-axis shows log2 fold change in the probability of accessibility of a 7mer (centered at the position indicated on the *x*-axis) between structures folded using the edited (G) vs. unedited (A) version of the sequences calculated using RNAplfold^46^ (see Methods). Shaded areas represent confidence intervals. Black bar indicates regions with a significant difference comparing the relative accessibilities around editing sites and random A’s (*p* value < 0.01, Wilcoxon Rank-Sum test). **b** Nucleotide frequencies opposite to the editing site in minimum free energy structures predicted with RNAfold^68^ using the unedited (blue bars) and edited sequences (red bars). ‘N’ is shown for structures where the editing site was unpaired and the opposite nucleotide was ambiguous (e.g., if the editing site was in a hairpin loop or bulge with multiple likely opposite nucleotides). **c** Correlation between relative 7mer accessibilities (calculated as in **a**) at predicted miRNA target sites (*x*-axis) and gene expression differences between edited (editing level ≥ 0.3) and non-edited individuals (*y*-axis, ratio calculated as edited/non-edited). Each circle represents average values of relative accessibility and expression ratio of a group of miRNA target sites (*x*-axis) and genes (*y*-axis) with similar distance (differing by ≤ 10 nt) between editing sites and miRNA target sites. Pearson correlation coefficient and significance of correlation are shown. **d** Scheme for the proposed structure-mediated regulation of target mRNA abundance depending on A-to-I editing

To understand the underlying mechanism of preferential targeting of unedited mRNA by AGO2-miRNA, we first considered the hypothesis that editing may alter sequences of miRNA target sites directly. However, we found that the editing sites of interest in this study rarely (2.2% of all sites) fell into miRNA seed target regions. Next, we asked whether RNA secondary structures were different between the edited and unedited sequences. We used RNAplfold^46^ to predict the accessibility (i.e., single-strandedness) of local RNA regions (7 nt windows) in the neighborhood of RNA editing sites (Methods). As the thermodynamic properties of inosines are not defined, previous studies have used guanosines, with closest properties to inosines, to replace inosines in RNA structure predictions^42^. Using this approach, we found that substituting A’s with G’s at the editing sites induced a significant decrease in accessibility near the edited A (Fig. 6a). This level of change is significantly larger than that at random A positions substituted by G’s (Fig. 6a).

On the basis of the RNAfold predictions, about half of the unedited A’s were opposite to a U nucleotide in the RNA structure folded using A’s at the editing sites (Fig. 6b). This opposite nucleotide was often (~ 55%) located within 100 nt from the editing site (Supplementary Fig. 10). The second most often observed nucleotide opposite to the unedited A was C (~ 15%), consistent with previous reports^47,48^. RNA structures folded using G’s at the editing sites were also enriched with C’s and U’s at the opposing positions, thus forming G-C or G-U pairing (together accounting for >80% of all structures). Thus, the edited nucleotides more often base-pair than the unedited nucleotides (80% vs. 52%). These results are consistent with the above observation of reduced accessibility (i.e., more double-stranded pairing) in the presence of G’s compared to A’s.

We next asked whether editing-induced structural changes correlate with altered miRNA target gene expression. We analyzed the expression difference of miRNA target genes between individuals with and those without edited sites in these genes. Interestingly, we observed a significant negative correlation between the target gene expression difference (expression ratio calculated as edited/unedited) and the relative accessibility at the closest miRNA target site (accessibility of G vs. A) (Fig. 6c, see Supplementary Data 1). It should be noted that the accessibility in Fig. 6c was calculated at miRNA target sites, most of which (84%) were located > 10 nt away from the corresponding editing sites. Thus, the relative accessibility varies in both positive and negative directions (see Fig. 6a for comparison). To reduce the impact of possible uncertainties in editing level estimation and secondary structure prediction, genes with similar distance (differing by ≤ 10 nt) between editing sites and miRNA target sites were grouped together and each group’s average values were shown in Fig. 6c. Altogether, our results suggest that A-to-I editing predominantly reduces miRNA accessibility (stabilizes the RNA secondary structure), which prevents AGO2-miRNAs from accessing its target sites and destabilizing the edited mRNAs (Fig. 6d).

### Experimental validation of editing-modulated mRNA abundance

**Fig. 7:**
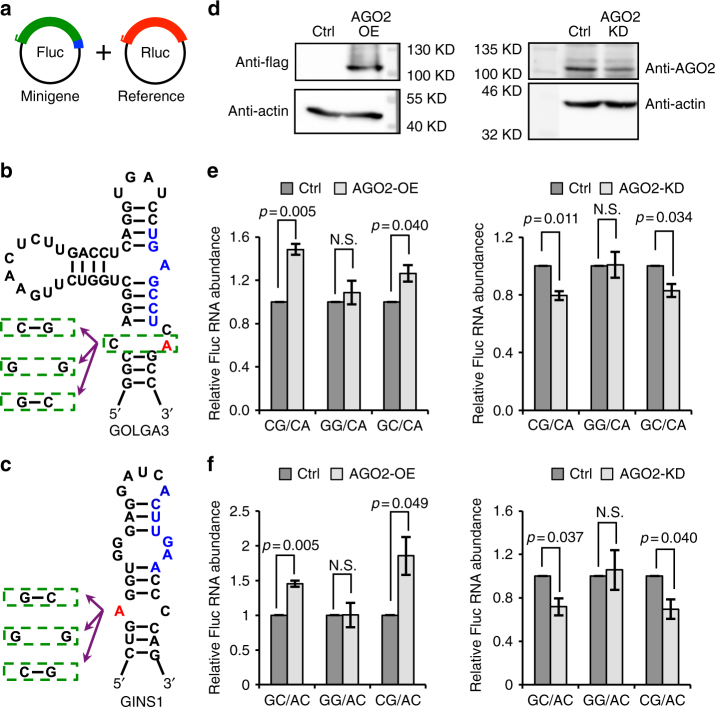
RNA editing-induced secondary structural changes affect RNA abundance. **a** Schematic diagram of the minigene system. Target sequences (3′ UTRs of *GOLGA3* and *GINS1*) and their mutant versions were inserted into the *firefly* luciferase (Fluc) 3′ UTR. *Renilla* luciferase (Rluc) was used as a reference reporter. Fluc and Rluc were co-transfected into HEK 293 control cells, *AGO2* overexpression (OE) and *AGO2* knockdown (KD) cells. RT-qPCR was carried out 48 h after transfection (details described in Methods). Green region represents *firefly* luciferase, red region represents *Renilla* luciferase and blue represents inserted target sequences or mutant versions. **b** Predicted RNA secondary structure of *GOLGA3* 3′ UTR flanking the RNA editing site. The “A” nucleotide at the editing site is highlighted in red. Blue sequences correspond to predicted miRNA target site (of the miRNA seed region). Green dashed boxes illustrate the unedited (A), pre-edited (G) and mutant nucleotides and their counterpart bases in the predicted RNA secondary structure, respectively. **c** Similar as **b**, but for the *GINS1* gene. **d** Western blot of AGO2 expression in control cells and cells with *AGO2* OE or KD. **e**, **f** Relative RNA expression levels of minigenes with unedited, pre-edited or mutant versions of the sequences shown in **b** and **c** respectively. Fluc and Rluc vectors were co-transfected into HEK293 control (Ctrl) cells and cells with *AGO2*-OE (left panel) or *AGO2*-KD (right panel). Relative Fluc RNA abundance between pre-edited (or mutant) and unedited versions of the minigenes is shown (see *x*-axis labels). Error bars represent standard deviation based on three experimental replicates. *P* values were calculated using Wilcoxon Rank-Sum test. *N.S*. not significant (*p* ≥ 0.05)

To confirm the above global findings, we experimentally tested two candidates, the *GOLGA3* and *GINS1* genes, which both contain RNA editing sites and miRNA target sites in their 3′ UTRs. These 3′ UTR regions were cloned into the 3′ UTR of a firefly luciferase minigene (Fig. 7a, Supplementary Table 2, Methods). The relative locations of RNA editing sites, miRNA target sites, as well as the predicted RNA secondary structures are illustrated in Fig. 7b, c. Both genes were confirmed as miRNA target genes (miR-24 targeting *GOLGA3* and miR-26 targeting *GINS1*) (Supplementary Fig. 11, Methods). For each minigene, we first constructed two versions—one harboring an “A” nucleotide at the RNA editing site and the other, a “G”—representing the unedited and edited 3′ UTRs, respectively. These constructs were then transfected into HEK293 cells for analyses.

For both candidates, we observed that the G version of the minigene had higher expression than the A version in cells that overexpressed (OE) *AGO2*, suggesting that the edited transcript (G version) is less prone to AGO2 regulation (Fig. 7d, e, left panel, CG/CA comparison; Fig. 7f, left panel, GC/AC comparison). Consistent with this observation, the G version of the minigene had lower expression than the A version in *AGO2* knockdown (KD) cells (Fig. 7d, e, right panel, CG/CA comparison; Fig. 7f, right panel, GC/AC comparison). The predicted RNA secondary structures showed that the unedited transcript had a CA mismatch at the editing site, whereas the edited version had a CG match (thus more stable) (Fig. 7b, c), which can explain the above results. Note that the above quantification was calculated relative to the CA or AC minigenes, to normalize against possible variability in controls due to variation in co-transfection efficiency, cellular condition, etc.

The observed differential expression of the A and G-harboring minigenes may be explained by two possible mechanisms: alteration of the miRNA binding sequence (beyond the seed target sequence) or RNA secondary structure, both of which may change AGO2-miRNA targeting of these genes. To investigate these possibilities, we constructed mutant versions of the minigenes with a GG mismatch in the predicted RNA secondary structure at the RNA editing site (Fig. 7b, c). We observed that RNA expression level of the GG minigene was similar to that of the original unedited version of the minigene (Fig. 7e, GG/CA comparison; Fig. 7f, GG/AC comparison) in both *AGO2* OE and KD cells. This observation suggests that the differential expression between unedited and pre-edited minigenes is likely due to changes in the RNA secondary structure, rather than sequence alterations to the miRNA binding sites. To further confirm this hypothesis, we created additional mutant minigenes with matches of identical base-pairing to the original edited minigene versions: GC for *GOLGA3* and CG for *GINS1*. Similar to the original edited minigenes, these mutant minigenes were also less prone to AGO2 regulation than the unedited versions (Fig. 7e, GC/CA comparison; Fig. 7f, CG/AC comparison). Thus, the above results suggest that the edited version of the transcripts is less responsive to AGO2 regulation, likely reflecting reduced miRNA accessibility resulting from stabilized RNA secondary structures. As a negative control, a similar experiment using a gene without predicted miRNA target sites in its 3′ UTR did not yield a difference in gene expression when substituting A with G at the editing site (Supplementary Fig. 12). These experimental data are consistent with the global analyses, suggesting that RNA structural changes due to editing explain the editing-dependent AGO2-miRNA targeting.

## Discussion

In this study, we carried out a global analysis of RNA editomes in human populations using RNA-Seq data of 462 individuals. The large amount of data afforded a global view of RNA editing profiles across human populations. In general, editing sites differ greatly in their prevalence in a population, ranging from being very rare to nearly 100% prevalent. Compared to rare sites, prevalent editing sites are stronger substrates for ADAR editing and are associated with higher editing levels. These observations hold true for all five populations included in this study. Indeed, our analyses showed that editing profiles of the five populations are largely similar, with small differences in editing levels of specific sites. This similarity in editing profiles strikingly contrasted the significantly different expression levels of the *ADAR* genes across populations.

To understand this apparent discrepancy, we conducted a series of in-depth analyses, supported by experimental validations, which led to the discovery of a new functional mechanism by which RNA editing can modulate mRNA abundance. Our results suggest that the AGO2-miRNA complex preferentially targets and destabilizes the unedited version of mRNAs that harbor editing sites in their 3′ UTRs. Furthermore, this differential regulation is likely mediated by a difference in RNA secondary structure caused by RNA editing. In general, the edited copy of an mRNA has a more stable secondary structure and renders lower accessibility for miRNA targeting than the unedited copy.

It has been a challenge in the field to better understand the functional impact of RNA editing, especially in human cells where most editing occurs in non-coding regions. Based on known examples of A-to-I editing sites affecting nuclear retention of mRNAs^16,17^ and inosine-specific degradation^18^, it was speculated that editing generally reduces the expression levels of its target genes. However, recent large-scale studies in brain revealed a comprehensive landscape of correlation between mRNA abundance and RNA editing levels (or *ADAR* expression levels), which consists of both positive and negative correlations^49,50^. These observations imply that RNA editing may affect mRNA abundance through a variety of mechanisms, which calls for systematic analyses to reveal such mechanisms.

Our study revealed one such mechanism where 3′ UTR-associated A-to-I editing affects mRNA degradation by modulating RNA secondary structure and accessibility for miRNAs at their target genes. It should be noted that a general stabilization of RNA secondary structure by A-to-I editing has been reported previously^47,48^. Our data suggested that the unedited versions of mRNAs are more prone to miRNA targeting, explaining the existence of positive correlation between mRNA abundance and RNA editing levels. Previously, ADAR and A-to-I editing were reported to affect miRNA biogenesis through different mechanisms, including editing in primary miRNA transcripts that may alter mature miRNA sequences, editing-dependent suppression of miRNA biogenesis steps, or editing-independent regulation due to interaction/competition between ADAR and miRNA biogenesis machineries^1,20,51^. In addition to affecting miRNA sequences or abundance, it is conceivable that A-to-I editing may alter the sequences of mRNA 3′ UTRs targeted by miRNAs. This hypothesis has been proposed repeatedly^26,29,30^, given that A-to-I editing and miRNA target sites both occur frequently in 3′ UTRs. However, computational analyses showed that RNA editing tends to avoid miRNA target sites^29,30^, which argues against this simple hypothesis. Thus, our results revealed a new mechanism that is different from previous speculations: miRNA targeting is affected by RNA structural changes mediated by A-to-I editing rather than direct alteration of miRNA target site sequences.

It should be noted that the endogenous gene expression changes caused by RNA editing are generally small in this study, which is likely due to the relatively low level of RNA editing for most sites (editing level being 0.1–0.2) and the fact that miRNA targeting mostly causes small to modest changes in mRNA expression levels^52^. Nevertheless, the collective changes exerted by many RNA editing sites together may be very influential to cellular function. In addition, such functional impact of RNA editing may be highly important under certain conditions, such as environmental stress or diseases, a subject for future investigations.

Our findings also revealed the complexity in the underlying mechanisms that account for the observed RNA editing variations. A conventional view of regulation of RNA editing levels focuses on direct mechanisms that influence ADAR-mediated RNA editing. Known mechanisms of such regulation include contributions by RBPs other than ADARs^53,54^, or genetic variations (e.g., single-nucleotide polymorphisms (SNPs)) that may affect the structural substrates of ADAR editing^55,56^. In contrast, our study suggests that the functional consequence of RNA editing (in affecting mRNA abundance) may in turn alter the observed editing levels. Editing-dependent changes in AGO2-miRNA targeting explain, at least partially, the apparent positive correlation between *AGO2* expression and RNA editing levels and the seemingly conflicting observations of large variations in *ADAR* expression and small variations in RNA editing levels across populations. Nevertheless, our study does not preclude the existence of additional mechanisms (such as those mediated by RBPs and SNPs) that may explain population-wide RNA editing similarity, which should be examined in the future. In addition, future studies are needed to investigate the underlying mechanism that accounts for the large variations in *ADAR* expression across populations.

Recently, an increasing number of studies revealed global or specific changes in RNA editing profiles in various human diseases or biological processes^8–12,49^. These studies indicate that RNA editing may have important functional implications in biology and disease. However, the functional consequence of the majority of non-coding editing sites remains elusive. Our study addresses this pressing question and provides insights into the functional impact of RNA editing in human cells.

## Methods

### Identification of RNA editing sites

Raw Fastq files of the Geuvadis RNA-Sequencing project^32^ were downloaded from the Geuvadis Data Browser (http://www.ebi.ac.uk/Tools/geuvadis-das) and mapped to the human genome (hg19) and Ensembl transcriptome^57^ using RASER^33^ with the parameters –m 0.05 –b 0.03. Potential mismatch positions were identified. A likelihood model was constructed to identify mismatch sites possibly due to sequencing errors. This model examines quality scores of the nucleotides, mismatch positions in the read and read coverage to calculate a log-likelihood ratio (LLR) for each mismatch site. Sites with LLR < 2 were removed as likely sequencing errors^5^. These mismatch sites were further filtered to require: (1) read coverage per site ≥ 10; (2) number of edited reads ≥ 2; and (3) editing level (ratio of edited to total reads) ≥ 0.1. Furthermore, mismatch sites were discarded if they overlapped known SNPs (dbSNP v141^58^), simple repeats (downloaded from UCSC table browser, genome.ucsc.edu), homopolymers of more than 4 nts, or located in introns within 4 nts of a splice junction (using UCSC and Ensembl gene annotations^57^)^6^. At last, we used GIREMI to call the final editing sites with the aim of distinguishing editing sites from unknown SNPs without requiring genome sequencing data^34^.

### Genomic context of editing sites

The genomic context of editing sites was determined using Ensembl gene annotations. If an editing site overlapped multiple types of genomic regions, its context definition was prioritized as follows: CDS > 3′ UTR > 5′ UTR > non-coding (nc)Exon > Intron-close > Intron-deep > Intergenic. ncExons refer to exonic regions in non-coding transcripts. Intronic editing sites were divided into those within 300 nucleotides and those that are further away from an exon-intron boundary (“Intron-close” and “Intron-deep”, respectively). The location of editing sites relative to Alu elements was determined using Alu repeats annotated by RepeatMasker^59^.

### Gene ontology analyses

Enriched gene ontology (GO) terms were obtained for 238 genes with prevalent editing sites compared to 722 genes with rare editing sites using PANTHER^60^. Selected GO terms were required to be significantly enriched (*p* < 0.05) with ≥ 2-fold enrichment among genes harboring prevalent editing sites, compared to the expected frequency determined using the background gene set (genes with rare editing sites). In addition, GO categories were discarded if < five genes were included. A similar analysis was conducted using genes with rare editing sites as the test set and those with prevalent editing sites as background.

### Test for shared and differential editing between populations

The significance of the number of shared edited sites between two populations was calculated using a hypergeometric test, with the background set being all 8080 editing sites considered in this study. Differentially edited sites were identified via a Wilcoxon Rank-Sum test comparing the editing levels of sites testable in two populations (*p* < 0.01). In addition, the absolute difference in average editing levels was required to be greater than 0.067, which corresponds to a difference of one edited read if the total read coverage was 15.

### Linear regression between editing levels and *ADAR* levels

Linear regression was carried out separately for every editing site, taking the editing level as the dependent variable and all three *ADAR* expression levels as regressors. Editing sites were considered if the number of individuals, in which the site was testable (covered by at least 10 reads), was larger than 10, and there were at least two different editing levels among these individuals. The least squares solution of the linear equation was computed using numpy.linalg.lstsq in Python2.7.

### Differentially expressed RBPs between populations

Differential expression analysis was performed using DESeq^61^ on a pair of populations treating individuals as replicates. Significantly differentially expressed RBPs were identified by an adjusted *p* value < 0.01. A list of 584 RBPs was compiled from different sources (ENCODE RBPs^62^, splicing factors^63–65^). Only RBPs with an average RPKM level > 1 across all individuals were considered.

### Predicted miRNA target sites and miRNA expression levels

Genomic coordinates of conserved miRNA target sites predicted by TargetScan5.1^44^ were downloaded from the UCSC Table Browser (genome.ucsc.edu). In addition, miRcode^45^ predicted target sites of conserved miRNAs were also included. For further analysis, we only included targets sites that were located in 3′ UTRs and restricted to predicted target genes of the 20 most highly expressed miRNAs in lymphoblastoid cell lines measured by small RNA-Seq (GEO accession code GSE41437)^66^. Normalized miRNA expression levels for 452 individuals were downloaded from ArrayExpress (http://www.ebi.ac.uk/arrayexpress/files/E-GEUV-3/GD452.MirnaQuantCount.1.2N.50FN.samplename.resk10.txt).

### miRNA targets in groups with different *ADAR1* or *AGO2* levels

To compare target gene expression between individuals with high and low *ADAR1*, but similar *AGO2*-miRNA levels (Fig. 5b), we first divided all individuals into three equally populated groups according to their *ADAR1* expression levels, and similarly for *AGO2* and miRNA expression levels, respectively. Subsequently, we compared gene expression levels between individuals with high and low *ADAR1* expression levels, controlling for *AGO2* and miRNA levels. The *AGO2* and miRNA expression levels of the two groups of individuals were required to be insignificantly different (*p* value > 0.1, Wilcoxon Rank-Sum test). This comparison was carried out separately for each miRNA and we required a minimum of 3 individuals in each group. The comparisons of gene expression levels between individuals with high and low *AGO2*-miRNA levels, but similar *ADAR1* levels (Fig. 5d), were carried out similarly.

### Analysis of *ADAR1* and *AGO2* knockdown and control experiments

Duplicated RNA-Seq data sets of *ADAR1* KD and control shRNA transfection in K562 and HepG2 cells, as well as *AGO2* KD and control shRNA transfection in K562 cells were downloaded from the ENCODE data repository (www.encodeproject.org) (accession numbers: ENCSR164TLB, ENCSR104OLN, and ENCSR495YSS, respectively). RNA-Seq reads were mapped using RASER similarly as described above for the Geuvadis data set. Gene expression levels (RPKM values^67^) were calculated using in-house scripts. Expression level of each gene in the two replicated samples of each condition was averaged first, followed by calculation of fold changes between groups of individuals. Editing sites in *AGO2* KD and control cells were identified as described above.

### Secondary structure predictions and related analyses

The RNA secondary structure was predicted for the region of ±500 nucleotides around an editing site using RNAfold^68^. RNAplfold^46^ was used to calculate the probability for accessibility of a 7mer in the same region using the parameters –u 7 -L 300 –W 400. The secondary structure and accessibility measure of the edited sequence was calculated by replacing the “A” with a “G” at the editing site. If multiple editing sites exist in the same 3′ UTR, each editing site was analyzed separately and included in the final result.

In Fig. 2b, the number of paired nucleotides in a sliding window of 9 nucleotides (sliding by 1 nucleotide) around editing sites or random A’s was calculated based on the minimum free energy structure predicted by RNAfold^68^ using the reference (unedited) sequence. The 9 nt window was centered at the position given on the *x*-axis.

The relative accessibility (Fig. 6a, c) was calculated as the log2 difference in accessibility of a 7mer at each position in the edited and reference structure (see Supplementary Software 1 and 2). In Fig. 6a, an average for each position was taken over all editing sites considered. Specifically, a sequence of length 1001 centered at the editing site was used as input to RNAplfold^46^. Then, for each position around the editing site, the probability for accessibility of a 7mer region in the RNA structure was calculated. This calculation was carried out for both the edited and unedited version of the 1001 nt sequence. The relative accessibility of a 7mer was calculated as the log2 difference in accessibilities at each position in the edited and unedited sequences (with editing site defined as position 0 and the 7mer centered at a given position). An average for each position was then taken over all editing sites considered in this analysis. In addition, as a control, the same calculation was done for random A nucleotides. Control random A’s were chosen to satisfy all of the following: (1) not editing sites, (2) located in 3′ UTRs, and (3) located 300 to 500 nt away from editing sites. We replaced such A’s with G’s and calculated the relative accessibility.

To directly analyze the consequences of editing-dependent structural changes at predicted miRNA sites (Fig. 6c), we compared the relative 7mer accessibilities at predicted miRNA target sites to target gene expression differences between individuals with editing levels ≥ 0.3 and individuals without edited reads, using the population data. We considered only the closest miRNA target site relative to each editing site, if multiple existed. In addition we considered only editing sites within genes of average expression level ≥ 0.1 and for which at least three edited and three non-edited individuals were available. Relative accessibilities and expression ratios were averaged over miRNA target sites and genes, respectively, with the closest distance between editing site and miRNA target site falling into the same sliding window of distances (window size 10 nt, sliding by 1 nt).

The nucleotides opposite to editing sites in the folded RNA structure (Fig. 6b) were identified in the minimum free energy structures calculated using RNAfold^68^. If the editing site was unpaired but one of its immediate neighbors was paired, the nucleotide next to the opposite nucleotide of the paired neighboring nucleotide was taken as the opposite nucleotide of the editing site. In all other cases where the editing site was unpaired, the opposite nucleotide was identified as ‘N’ (see Supplementary Software).

### Plasmid construction

Partial 3′ UTRs of *GOLGA3*, *GINS1*, and *C1GALT1* were inserted into the 3′ UTR of the *firefly* luciferase reporter via Sac I and Xba I cloning sites (see Supplementary Table 2 for inserted sequences). To make miRNA sponge vectors, three copies of miR-24 and miR-26 target sequences were inserted into the 3′ UTR of a GFP reporter via EcoRI and BamH I cloning sites (Supplementary Table 2). *AGO2* shRNA was inserted into the pLVX–U6-puro vector via the BamH I and EcoR I sites. Human *AGO2* cDNA with 3 × Flag tags at the N terminal (3 × Flag-hAGO2) was cloned into the pLVX-Tight-puro vector via BamH I and Not I sites.

### Lentiviral *AGO2* knockdown, overexpression and cell culture

To make lentivirus, control or *AGO2* shRNA plasmids or 3 × Flag-hAGO2 plasmids were co-transfected into 293T cells (ATCC, CRL-3216) with pΔ8.9 and pVSV-G in ratio 4:3:2. pTet-on Advanced and pMD2.G vectors were co-transfected into GP2-293 cells (ClonTech, 631458) in ratio 1:1 to produce Tet-on retrovirus. All viruses were collected at 48 h after transfection. To produce Tet-on HEK293 cells, HEK293 cells (ATCC, CRL-1573) were infected by the Tet-on retrovirus for 48 h followed by 500 μg per ml G418 selection. Then HEK293 cells were infected by the control or *AGO2* shRNA lentivirus, and HEK293-Tet-on cells were infected by the control or 3 × Flag-hAGO2 lentivirus for 48 h followed by 2 μg per ml puromycin selection.

HEK293 cells were cultured with DMEM medium containing 10% FBS. HEK293 cells with *AGO2* knockdown were cultured with DMEM medium containing 10% FBS and 0.5 μg per ml puromycin. HEK293 cells with *AGO2* overexpression were cultured with DMEM medium cantaining 10% FBS, 0.5 μg per ml puromycin and 100 μg per ml G418.

### Transfection

A total of 2.5 μg miR-24 or miR-26 sponge vector were transfected into HEK293 cells. After 24 h of transfection, 2.4 μg sponge, 50 ng reporter and 50 ng reference vector were co-transfected into pre-neutralized HEK293 cells. Cells were collected at 48 h after transfection. Control, *AGO2* knockdown and overexpression cells were plated into 6-well plates 1 day before the experiment. *AGO2* overexpression was induced by 2 μg per ml doxycycline. Then 50 ng reporter and 50 ng reference plasmids (*Renilla* luciferase) were co-transfected into cells. Cells were collected 48 h after transfection.

### Western blot

*AGO2* overexpression was induced by 2 μg per ml doxycycline. Cells were lysed in RIPA buffer and protease inhibitor (ThermoFisher, 88266) 48 h after induction, and the total cell lysates were resolved with SDS-PAGE gels. The following antibodies were used: Flag antibody (sc-807, at 1:3000 dilution) and beta-actin antibody (sc-47778, at 1:3000 dilution) from Santa Cruz, AGO2 antibody (ab156870, at 1:2000 dilution) from Abcam. The HRP-linked secondary antibodies from Santa Cruz (goat anti-rabbit IgG-HRP: sc-2004, goat anti-mouse IgG-HRP: sc-2005, both at 1:2000 dilution) were used and the blots were visualized with the ECL kit (GE, RPN2232). The uncropped Western blot images for AGO2 OE and AGO2 KD with controls are shown in Supplementary Fig. 13.

### RNA isolation and Real-time qPCR

Total RNA was isolated using TRIZOL reagent and treated with DNase I (37° C, 1 hr, followed by heat inactivation). A total of 2 μg total RNA was reverse-transcribed using SuperScript III (Invitrogen). The qPCR was performed using the PowerUp™ SYBR® Green Master Mix (Thermo Scientific) and a LightCycler 480 system (Roche) according to manufacturer’s instructions.

### Code availability

Computer scripts used to analyze RNA secondary structures and accessibility are included as Supplementary Software 1 and 2.

### Data availability

All data sets used in this study are publicly available. Raw RNA sequencing data (paired-end sequencing of polyA-selected RNA) for 462 individuals of the Geuvadis RNA-Seq project^32^ were downloaded from http://www.ebi.ac.uk/ena/data/view/PRJEB3366. RNA-Seq data sets of *ADAR1* KD, *AGO2* KD and corresponding control data sets were downloaded from the ENCODE data repository (www.encodeproject.org; accession numbers: ENCSR164TLB, ENCSR104OLN, and ENCSR495YSS). Normalized miRNA expression levels for 452 individuals were downloaded from ArrayExpress (http://www.ebi.ac.uk/arrayexpress/files/E-GEUV-3/GD452.MirnaQuantCount.1.2N.50FN.samplename.resk10.txt). Read counts of miRNAs in lymphoblastoid cell lines measured by small RNA-Seq were downloaded from GEO (www.ncbi.nlm.nih.gov/geo/; accession code GSE41437)^66^.

## Electronic supplementary material

Supplementary Information

Description of Additional Supplementary Files

Supplementary Data 1

Supplementary Software 1

Supplementary Software 2
