# Supplementary material for: Structure-mediated modulation of mRNA abundance by A-to-I editing
Source: Nat Commun. 2017 Nov 2;8:1255. doi: 10.1038/s41467-017-01459-7 (PMC5665907; doi:10.1038/s41467-017-01459-7)
Supplement: Supplementary file 1 — Supplementary Information [file 41467_2017_1459_MOESM1_ESM.pdf]

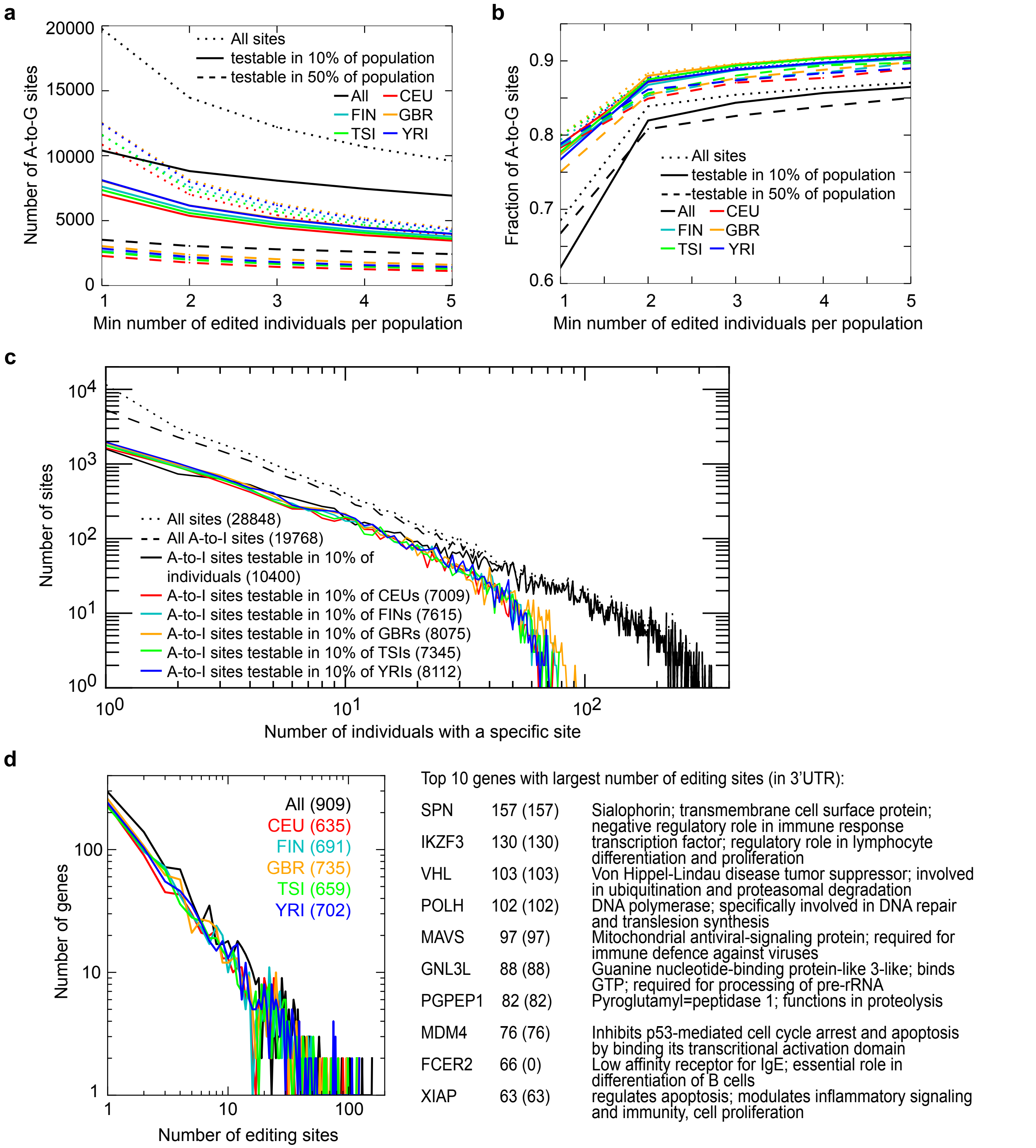

Supplementary Figure 1. Selection of highly confident A-to-I editing sites and distribution of editing sites among genes.

(a) Total number of RNA-DNA mismatches at different cutoffs of the required minimum number of edited individuals in a population. (b) Similar as (a), but for the fraction of A-to-I editing sites. (c) Number of sites shared between indicated number of individuals (x-axis) for all types of RNA-DNA mismatches (dotted line), all A-to-I editing sites (dashed line), and A-to-I sites that were testable in 10% of all populations combined (black solid line), or any one population (colored lines). (d) Number of A-to-I editing sites per gene. Editing sites were required to be testable in 10% of the individuals in a population and edited in at least 3 individuals. The total number of genes with such editing sites is shown in parenthesis. The top 10 genes with the highest number of editing sites are shown, with the total number of editing sites and the number of editing sites in 3' UTRs (parentheses) indicated.

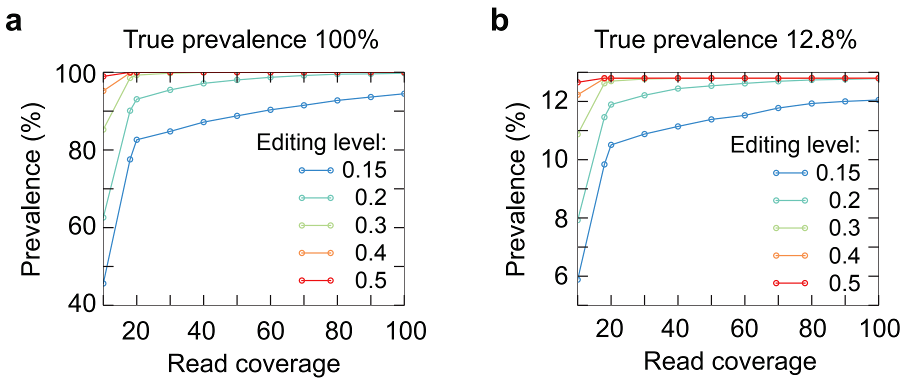

Supplementary Figure 2. Simulations to analyze the impact of read coverage on the observed prevalence of editing sites.

(a) Editing sites were simulated by assuming different editing levels as shown, and different total read coverage (x-axis). For each editing site, 500 individuals were simulated with 100% prevalence. The binomial distribution was used with the expected probability of success being the simulated editing level, to mimic the random sampling process in RNA-Seq. The figure shows observed prevalence (percentage of individuals with editing sites among all individuals) at different read coverage levels. The observed prevalence values were averaged over 100 simulated editing sites with their true prevalence being 100%. Based on this plot, the observed prevalence for 15% editing level (equivalent to the observed average editing level of "rare" sites in our data) is relatively low compared to sites with higher editing levels, especially at low total read coverage. At a total read coverage of 18 (average read coverage of rare sites in this study) for a 15% editing level, the observed prevalence is about 77%, an under-estimation of the true prevalence (100%). (b) Same as (a), but the true prevalence is simulated to be 12.8%, in order to achieve an observed prevalence of ~10% for editing level 0.15 and a read coverage of 18 reads (similar to the observed average editing level and read coverage of "rare" sites in our data). Thus, this result suggests that the true prevalence of rare sites defined in our study is indeed relatively low (about 12.8%).

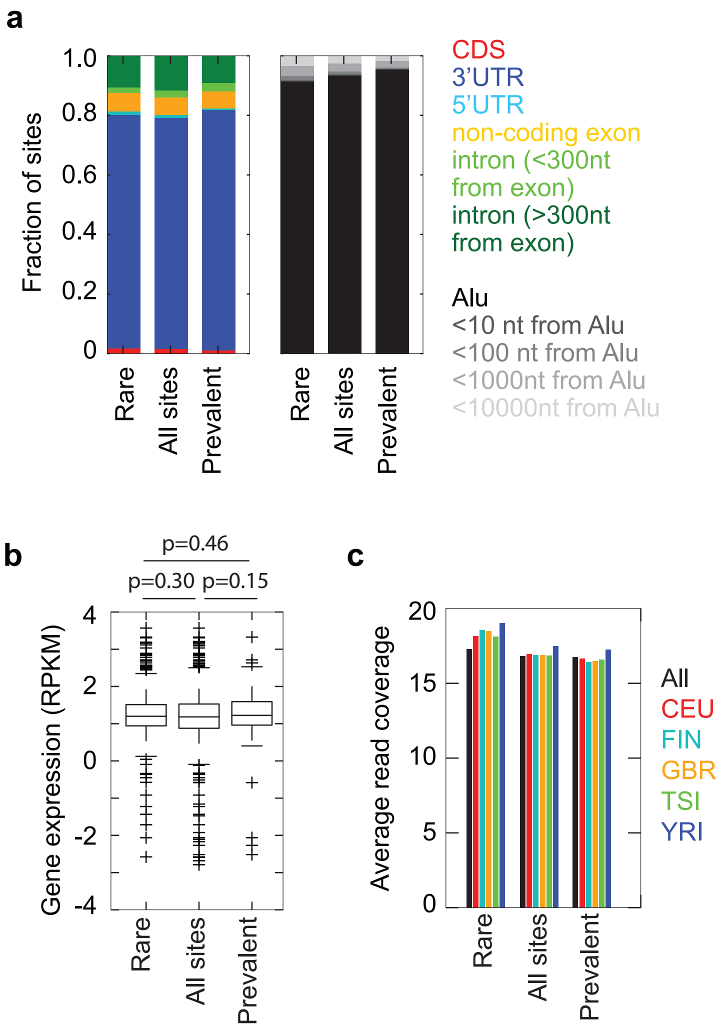

Supplementary Figure 3. Comparison of prevalent and rare editing sites.
(a) Distribution of editing sites in different types of genomic regions for rare (prevalence ≤ 10%, 3618 sites), prevalent (prevalence > 90%, 714 sites) and all editing sites (8080 sites).
(b) Expression levels of genes harboring rare, prevalent and all editing sites (p value calculated using Wilcoxon Rank-Sum test). (c) Read coverage at rare, prevalent and all editing sites.

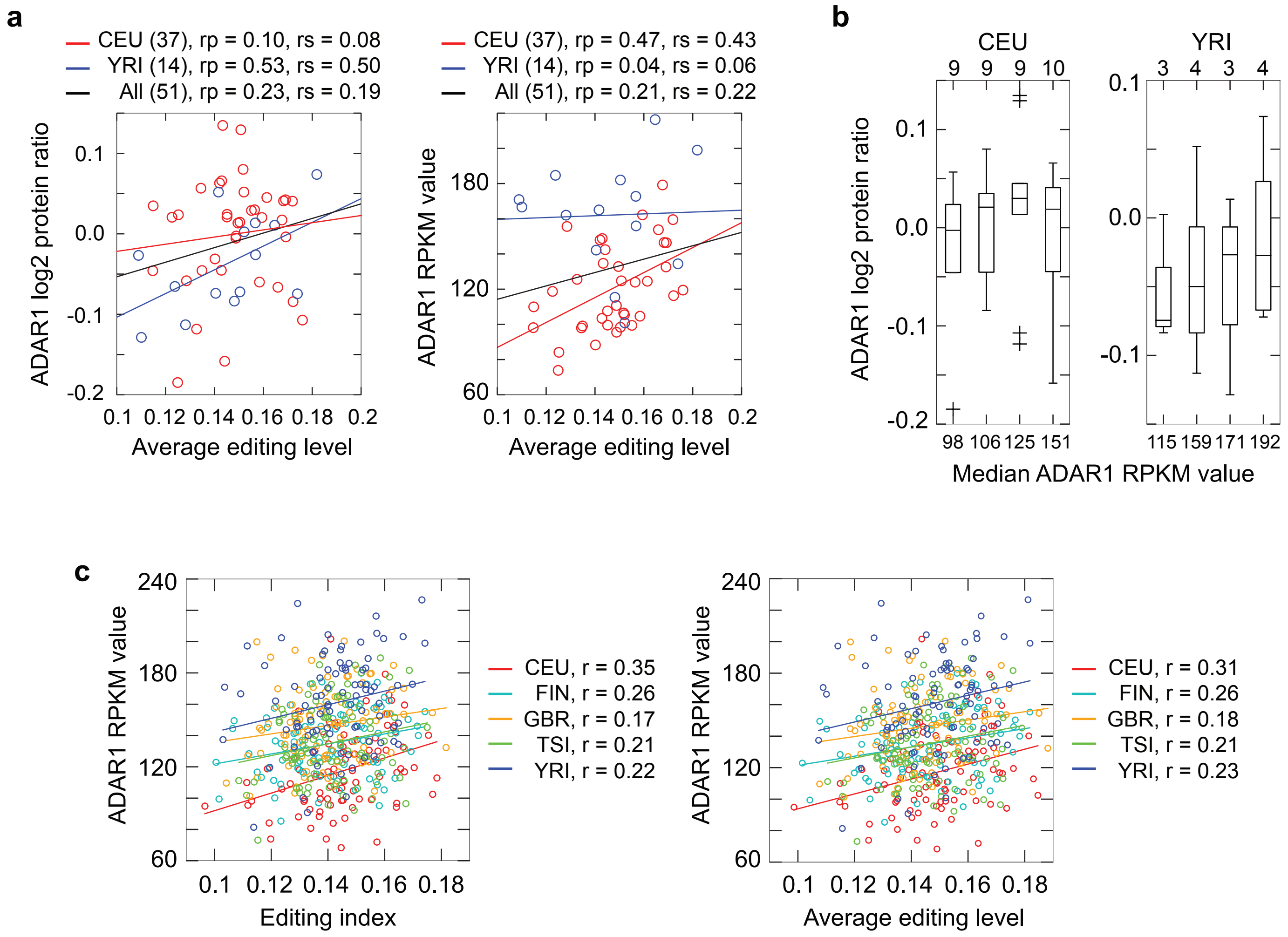

Supplementary Figure 4. Relationship between ADAR1 mRNA and protein levels and editing levels.

(a) Correlations between average editing level (per individual) and ADAR1 protein level (left) or ADAR1 mRNA levels (right). Included are 37 CEU and 14 YRI individuals that overlapped individuals for which protein levels were quantified (Wu et al. 2013). Correlation coefficients (rp: Pearson correlation coefficient, rs: Spearman correlation coefficient) were calculated for only CEU, only YRI, and for all individuals. (b) Relationship between ADAR1 mRNA levels (RPKM values) and ADAR1 protein levels (quantified as a normalized log2 protein ratio by Wu et al. Nature 2013) for 37 CEU individuals (left panel) and 14 YRI individuals (right panel). The correlation coefficients are 0.172 for CEU and 0.296 for YRI. The individuals are separated into 4 groups of similar size (number of individuals shown on top). (c) Correlation of editing index (left) or average editing level (right) with ADAR1 RPKM level for all populations.

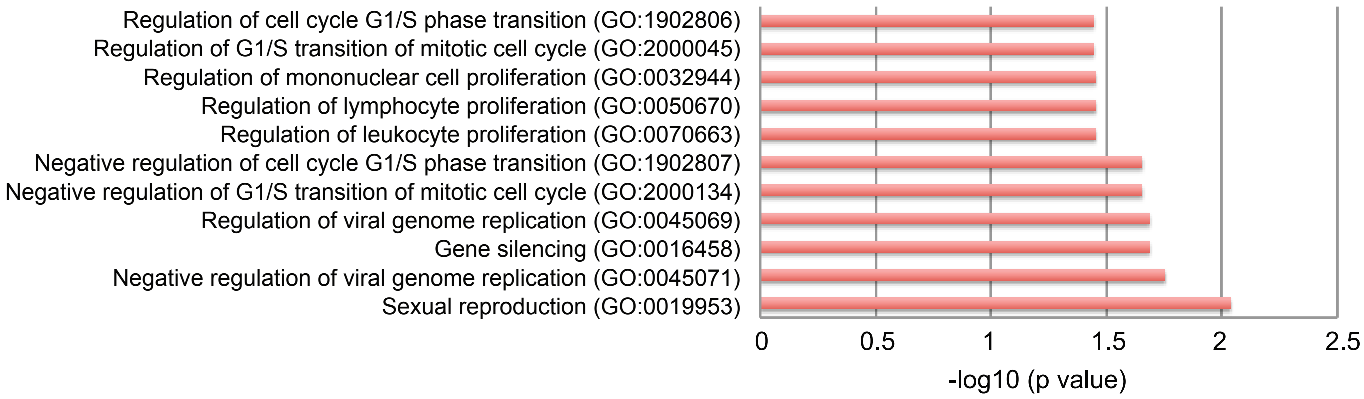

Supplementary Figure 5. GO term enrichment for genes with prevalent editing sites. Enriched GO terms among genes harboring prevalent editing sites compared to genes with rare editing sites ($p < 0.05$, fold enrichment >2,at least 5 genes in each GO term).

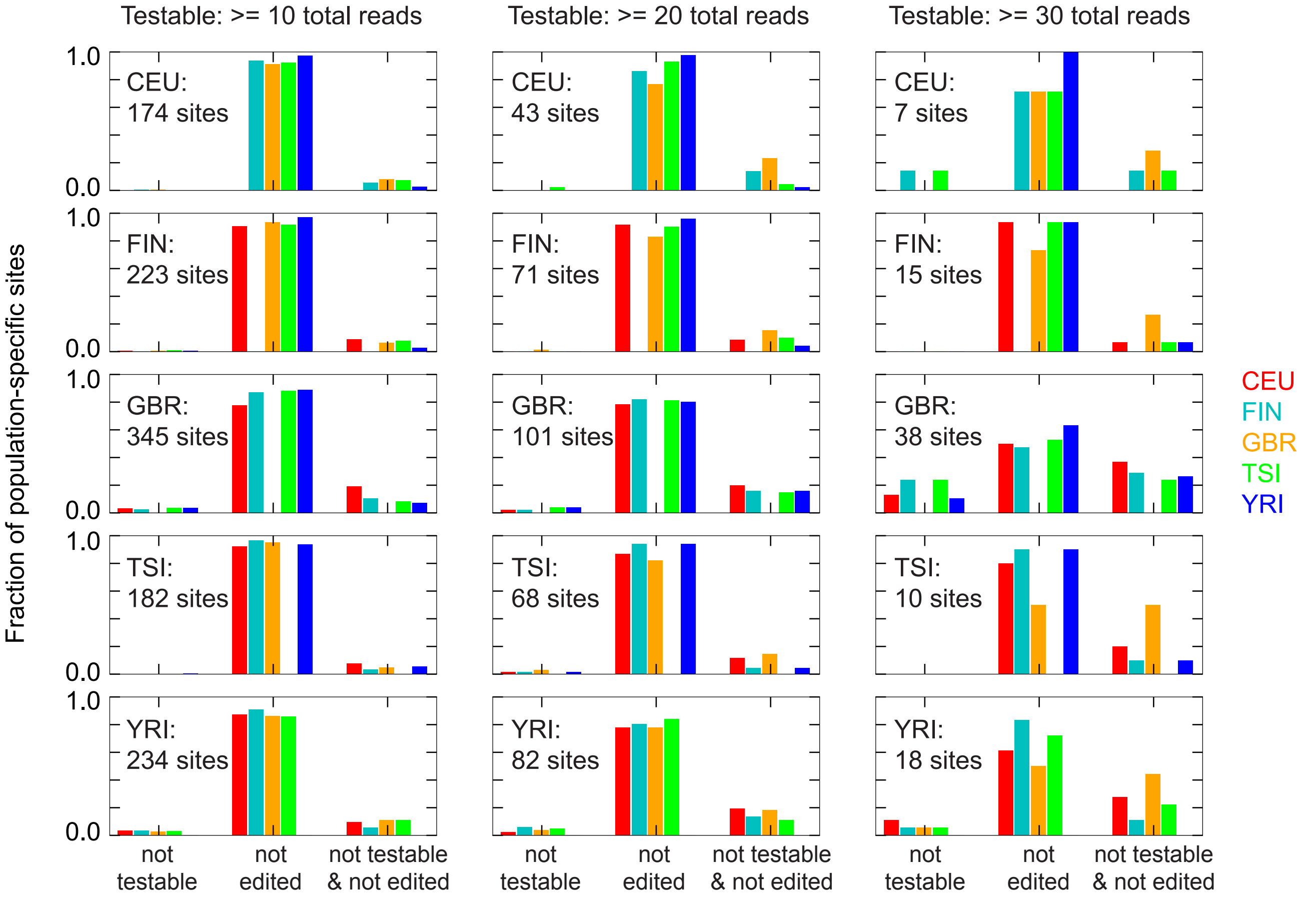

Supplementary Figure 6. Status of population-specific editing sites in other populations. Population-specific editing sites (1st row: CEU sites, 2nd row: FIN sites, 3rd row: GBR sites, 4th row: TSI sites, 5th row: YRI sites) in other populations are shown using color-coded bars. Population-specific sites are identified for 3 different read coverage requirements: ≥10 total reads per site (left), ≥20 total reads (middle) and ≥30 total reads (right). "not testable" indicates that the editing site did not pass the read coverage requirement: total number of reads passing the indicated threshold in ≥10% of the individuals of a population, "not edited" indicates that the editing site was testable, but not edited (edited defined as ≥2 edited reads and editing level ≥0.1 in at least 3 individuals of a population), and "not testable & not edited" indicates that the editing site failed being testable and edited in a population.

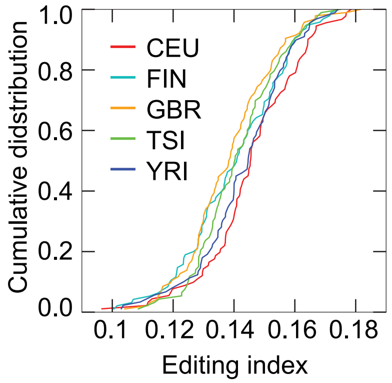

Supplementary Figure 7. Cumulative distributions of editing indexes in different populations.

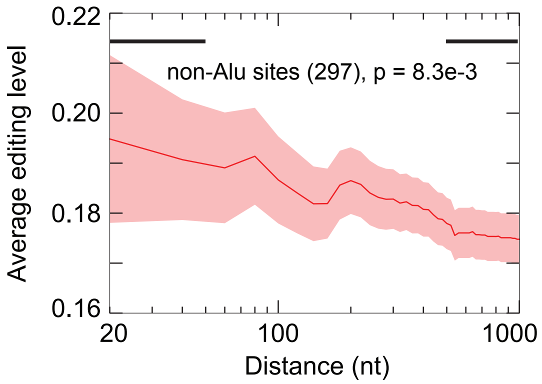

Supplementary Figure 8. Average editing levels in dependence of the distance to the closest predicted miRNA target site.

Average editing level in dependence of the distance between an editing site and a predicted miRNA target site for editing sites located in non-Alu regions. Shaded areas represent standard error of the mean. P values are shown to compare editing level difference at editing sites close (<50 nt, black bar at top) and relatively far (500-1000 nt, black bar at top) from predicted miRNA target sites (Wilcoxon Rank-Sum test).

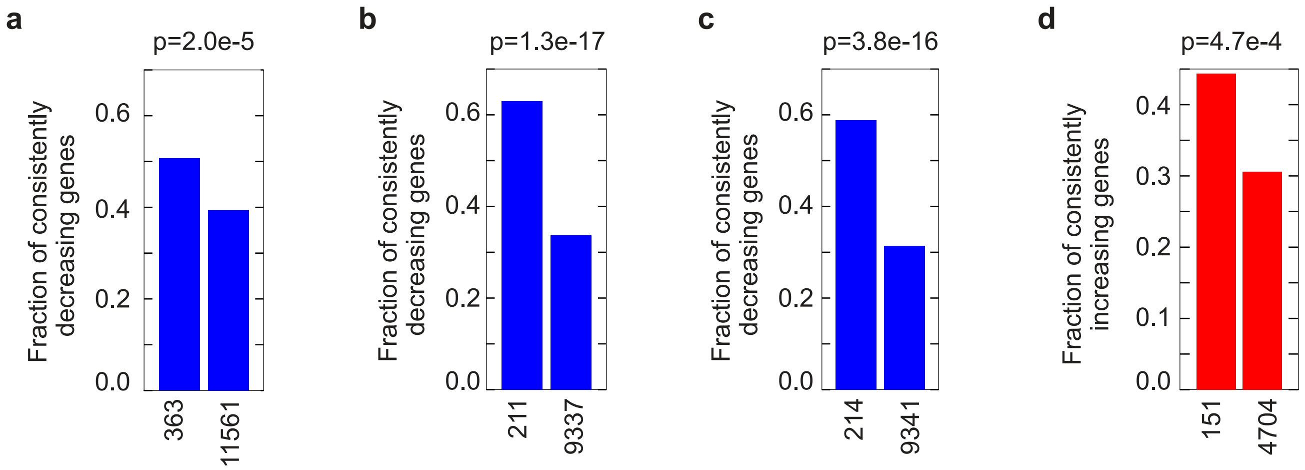

Supplementary Figure 9. Comparison of gene expression changes in ENCODE and 1000 genomes data sets.

(a) Genes with lower expression (log2 fold change <0) in ADAR1 KD (vs. control) in K562 cells compared to HepG2 cells. Genes shared between the two groups are counted. The fraction of such genes among all testable genes between the two data sets is calculated and shown on the y-axis. The first bar shows the results for genes with editing sites and miRNA target sites within 500nt. The second bar shows control genes that do not have editing sites in the 3' UTR. The total number of testable genes is shown below each bar.P value was calculated via Fisher's exact test. (b) Similar as (a), but for comparing genes with lower expression (log2 fold change <0) in ADAR1 KD (vs. control) in K562 cells and genes with lower expression in individuals with low ADAR1 levels (vs. those with high ADAR1 levels, shown in Fig. 5b, "AGO2 & miRNA high" group). (c) Similar as (b), for HepG2 cells. (d) Similar as (a), but analyzing genes with higher expression (log2 fold change >0) in AGO2 KD data and in individuals with low vs. high AGO2 expression levels (those genes shown in Fig. 5d, "ADAR1 low" group). The control genes consist of those without editing sites in the 3' UTR, but with miRNA target sites.

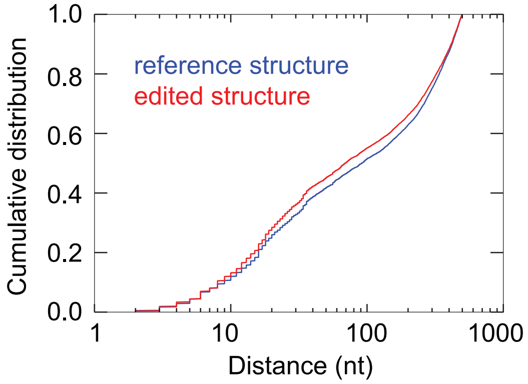

Supplementary Figure 10. Linear distance between editing site and its opposite nucleotide in the minimum free energy structure calculated by RNAfold.
The RNA structures were predicted using sequences +/- 500 nts around the editing sites. The structure for the edited sequence was calculated by replacing 'A' with 'G' in the reference sequence at the editing site.

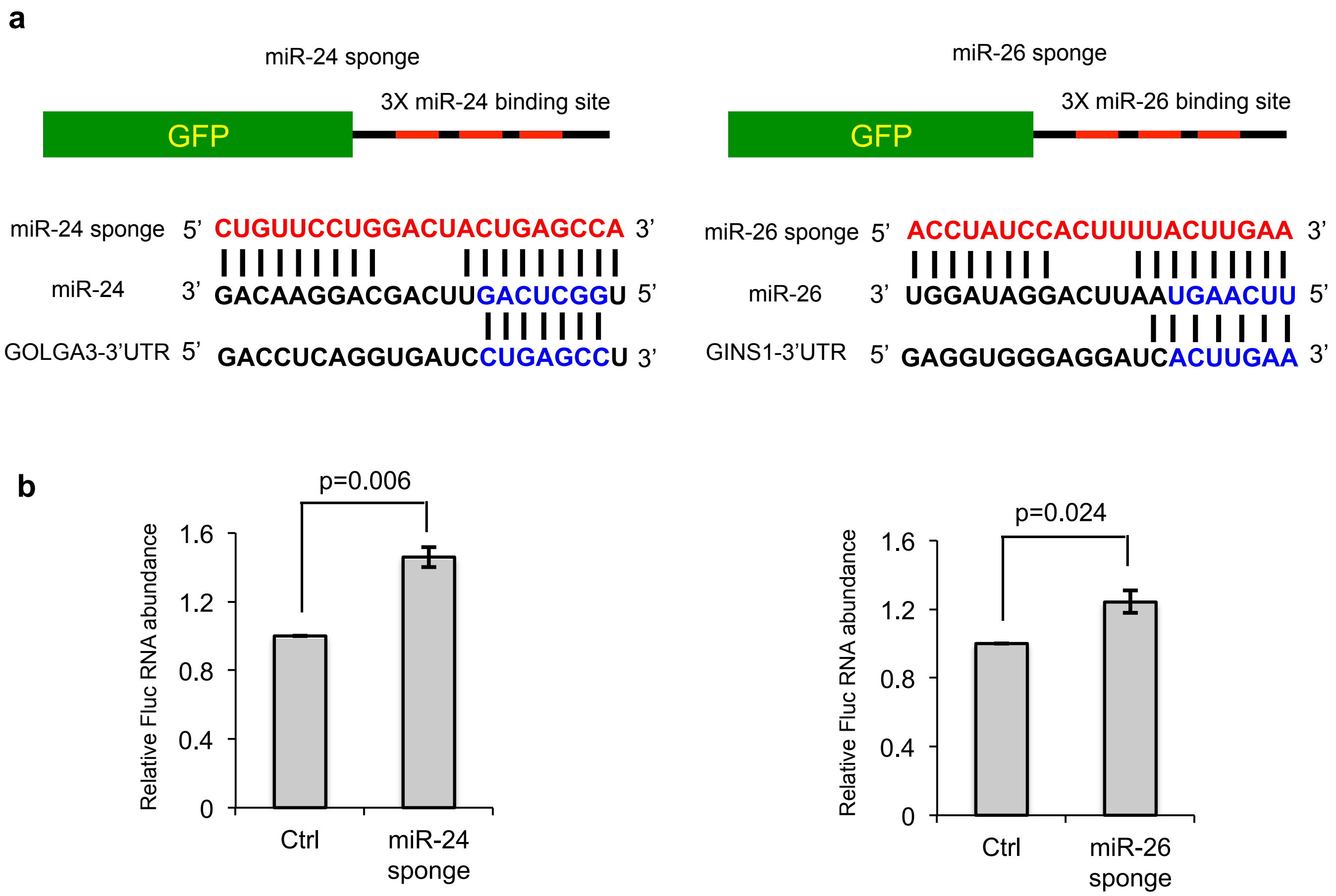

Supplementary Figure 11. Confirmation of microRNA targeting.
(a) Schematic diagram of microRNA sponges. Three copies of miR-24 or miR-26 bulged binding sites were inserted into the 3'UTR of eGFP. Red sequences represent miR-24 or miR-26 bulged binding sites. Blue sequences represent base-pairing between miR-24 and miR-26 seed region and target gene. (b) Fluc minigene expression level is increased if co-transfected with mircoRNA sponge. microRNA sponges or control vectors (Ctrl) are co-transfected into HEK293 cells with reporter genes. RT-qPCR was assayed 48hrs after transfection (please see details in Methods).

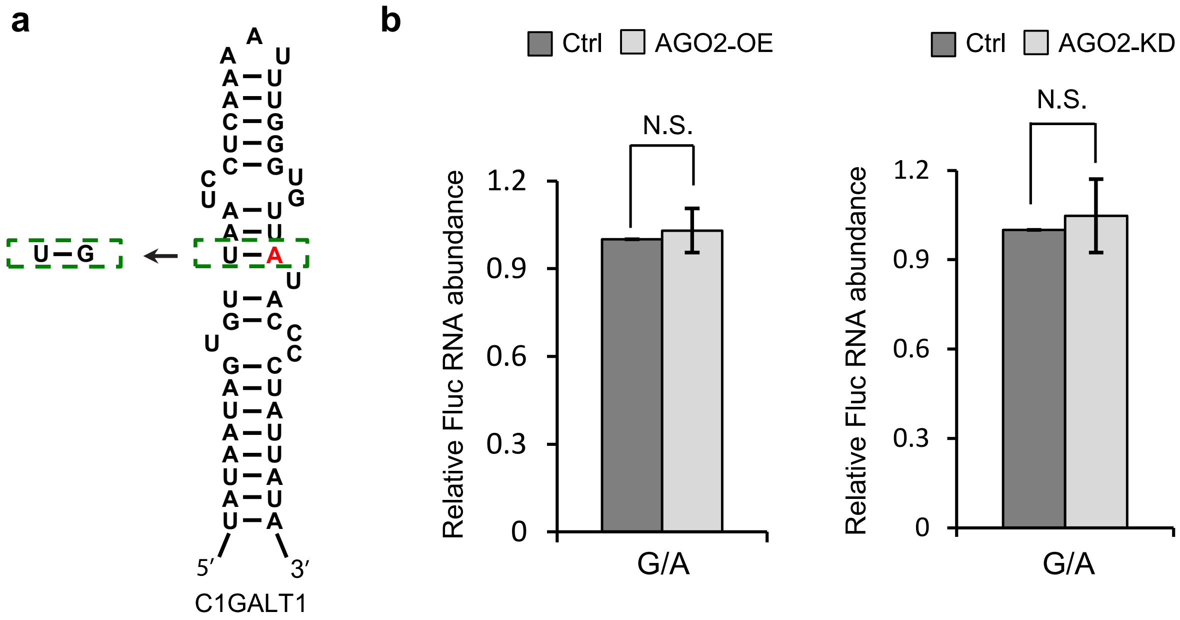

Supplementary Figure 12. An example negative control gene without predicted miRNA target sites.

(a) Predicted RNA secondary structure of C1GALT1 3' UTR by Mfold (Zucker 2003). The RNA editing site is shown in red. We made minigenes with the unedited (U-A in green dashed box) or edited (U-G in green dashed box) nucleotides respectively. See Methods for details.

(b) Relative Fluc RNA abundance of pre-edited (G) and unedited (A) nucleotide at the editing site, quantified by qPCR in AGO2 overexpression (left) and AGO2 knockdown (right).

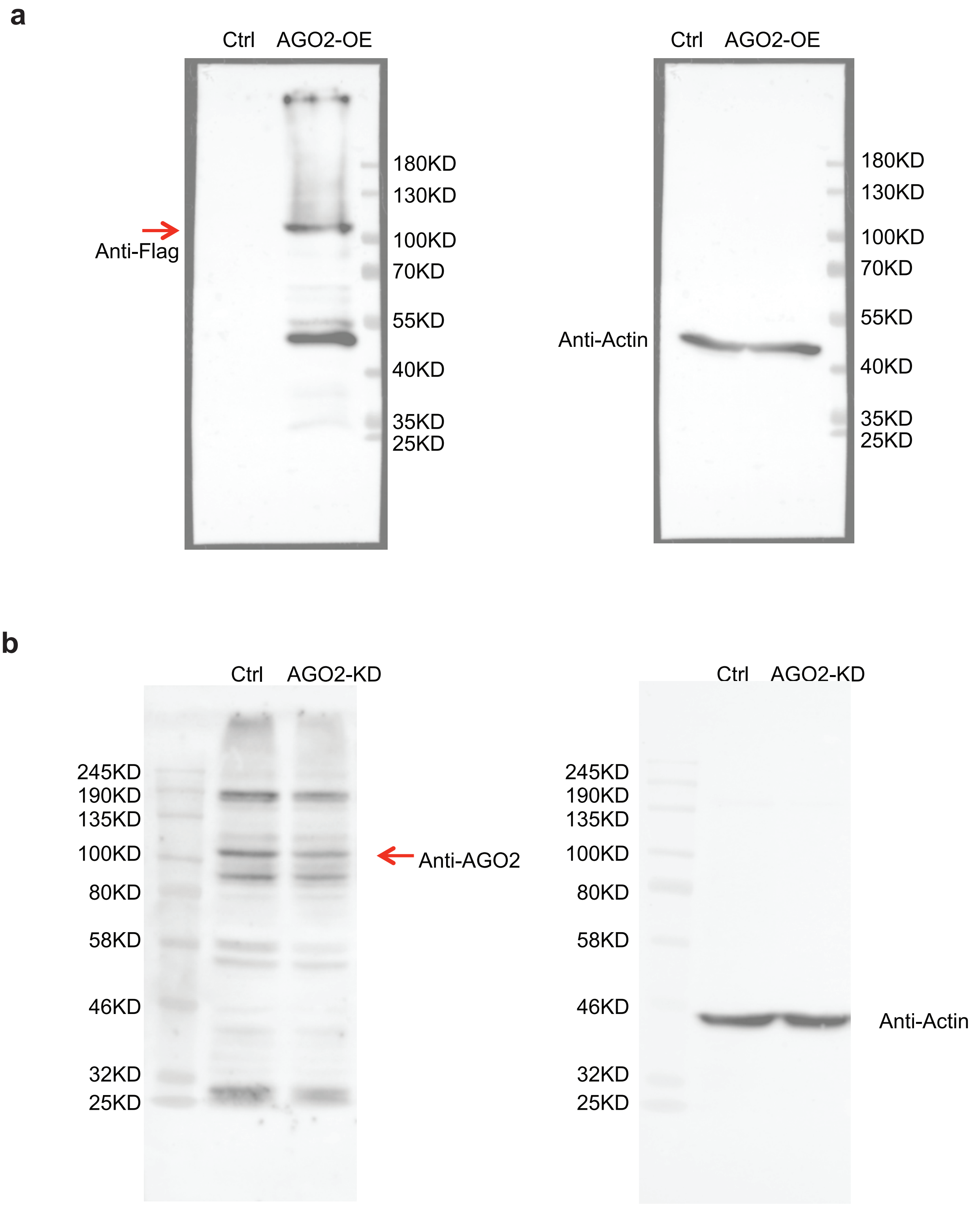

Supplementary Figure 13. Original Western blot images for AGO2 overexpression and knockdown experiments (corresponding to Fig. 7d)
(a) AGO2 expression in flag-AGO2 overexpression (AGO2-OE) cells and negative control cells. The red arrow indicates the over-expressed AGO2. Bands below AGO2 are degraded AGO2 fragments. Actin was used as a positive control. Protein marker is visualized using PageRuler™ Prestained Protein Ladder from ThermoFisher (26616).(b) AGO2 expression in sh-AGO2 KD and sh-control cells. The red arrow indicates the AGO2 protein. The top band is non-specific and bands below AGO2 are degraded AGO2 fragments and non-specific bands. Actin was used as a positive control. Protein marker is visualized using Color Prestained Protein Standard, Broad Range from NEB (P7712S).

Supplementary Table 1. Differentially expressed RNA binding proteins (RBPs; adjusted p value < 0.01) in at least 4 pairwise comparisons between populations. Log2 fold changes and adjusted p values are calculated using DESeq (Anders and Huber, 2010). The fold change is calculated for the pair of populations indicated, in particular second population/first population.

RBP / population comparison	Log2 fold change	Adjusted p value
**HNRNPLL**		
GBR_CEU	0.444689815	0.001615085
GBR_FIN	0.640365794	9.27E-06
TSI_CEU	0.843464719	8.46E-12
TSI_FIN	1.039140698	1.56E-15
YRI_CEU	0.505882385	0.000151444
YRI_FIN	0.701558363	2.65E-07
**IGF2BP3**		
FIN_CEU	0.931086162	1.14E-14
GBR_CEU	0.645222836	4.89E-08
TSI_CEU	0.742443025	6.03E-09
YRI_FIN	-0.82246351	2.63E-11
YRI_GBR	-0.536600184	1.13E-05
YRI_TSI	-0.633820373	3.82E-06
**AGO2**		
FIN_CEU	-0.499448837	6.95E-05
YRI_CEU	-0.881319439	8.96E-13
YRI_GBR	-0.711489287	1.79E-08
YRI_TSI	-0.641990404	1.68E-06
**EEF1A1**		
YRI_CEU	0.535438173	2.81E-05
YRI_FIN	0.4424296	0.000807285
YRI_GBR	0.419081832	0.001404191
YRI_TSI	0.421574392	0.001412327
**IGF2BP1**		
FIN_CEU	1.94632092	1.61E-06
GBR_CEU	1.563109282	0.000181513
TSI_CEU	1.460890123	0.001032272
YRI_FIN	-1.527126407	0.000537059
**LARP7**		
YRI_CEU	0.441065868	0.001262641
YRI_FIN	0.400548187	0.00683429
YRI_GBR	0.403719222	0.004473215
YRI_TSI	0.420003708	0.003998697
**LUC7L3**		
YRI_CEU	0.495894686	0.000359785
YRI_FIN	0.433694295	0.002452589
YRI_GBR	0.482381514	0.000363211
YRI_TSI	0.441396738	0.003393382

**MBNL2**		
FIN_CEU	-0.662310864	3.40E-08
GBR_CEU	-0.393103087	0.00204398
TSI_CEU	-0.462566222	0.000405465
YRI_CEU	-0.396905806	0.001809899
**PNN**		
YRI_CEU	0.425788529	0.003788712
YRI_FIN	0.42497142	0.003947445
YRI_GBR	0.476169988	0.000443548
YRI_TSI	0.422812149	0.004001926
**PPIL3**		
YRI_CEU	0.647310248	9.22E-07
YRI_FIN	0.605073309	1.06E-05
YRI_GBR	0.578854075	1.91E-05
YRI_TSI	0.542378482	0.00027228
**RBPMS2**		
FIN_CEU	1.050125846	0.002304445
YRI_FIN	-1.641706359	3.08E-08
YRI_GBR	-1.408812402	2.20E-06
YRI_TSI	-0.995579406	0.00586514
**SNRPA1**		
YRI_CEU	0.41626643	0.00300229
YRI_FIN	0.451729135	0.000926237
YRI_GBR	0.549158615	1.91E-05
YRI_TSI	0.425634441	0.004059557
**TRIM56**		
YRI_CEU	-0.562990466	2.09E-05
YRI_FIN	-0.485286936	0.001006385
YRI_GBR	-0.442454845	0.001908573
YRI_TSI	-0.535091267	8.09E-05

Supplementary Table 2:

(a) Primer sequences

Name	sequence(5'-3')	Note
GOLGA3-fwd2	CACGCGGACACTTCCTTCC	primer pair used for amplifing GOLGA3 3'UTR
GOLGA3-rev2	ACTCTGGAAGGTAGAAGTGC	
GOLGA3-sac-fwd	acgtgagctcACCATGTTGGCCAGGCTGG	primer pair used for cloning GOLGA3 3' UTR into the minigene
GOLGA3-xba-rev	gattctagaAAATCCAACTGCAGCCGGG	
GOLGA3-G-fwd	CCTGAGCCTCGGCCTCCC	primers used for making point mutation in GOLGA3 3'UTR
GOLGA3-G-rev	GGGAGGCCGAGGCTCAGG	
GOLGA3-sac-fwd2	acgtgagctcACCATGTTGGCGAGGCTGGTC	
GOLGA3-C-fwd	CCTGAGCCTCCGCCTCCC	
GOLGA3-C-rev	GGGAGGCGGAGGCTCAGG	
GINS1-fwd	CTCTCCTGAATGCCAATACC	primer pair used for amplifing GINS1 3'UTR
GINS1-rev	CAATCAAGGTGCCCAACAGG	
GINS1-sac-fwd	acgtgagctcGCCTATGGCAAAACTCCGTC	primer pair used for cloning GINS1 3' UTR into the minigene
GINS1-xba-rev	gattctagaTGGTGTGATCTCAGCTCACTG	
GINS1-G-fwd	GCAGGCTGGGGTGGGAGG	primers used for making point mutation in GOLGA3 3'UTR
GINS1-G-rev	CCTCCCACCCCAGCCTGC	
GINS1-G-rev2	CTCACTGCAGTCTTGACCTCTGCGGTTCAAG	
GINS1-C-fwd	GCAGGCTGCGGTGGGAGG	
GINS1-C-rev	CCTCCCACCGCAGCCTGC	
C1GALT1-fwd-Sac	acgtgagctcAATGCCAACTGTATGCTAGG	primer pair used for cloning C1GALT1 3' UTR into the minigene
C1GALT1-rev-Xba	gattctagaATCCTACCTCTGTTACTTAC	
C1GALT1-G-fwd	TTGGGTGTTGTACCCCTATTATAGATAAGG	primers used for making point mutation in C1GALT1 3'UTR
C1GALT1-G-rev	TAGGGGTACAACACCCAAATTTTGAGG	
C1-stop-fwd	TCGAGCTtagtgataaCA	primer pair used for insertion of stop codon at pEGFP-C1 N terminal
C1-stop-rev	AGCTTGTTATCACTAAGC	
Sponge26-fwd	cgcgaattcACCTATCCACTTTTACTTGAActgacACCTATCCACTTTTACTTGAA	primer pair used for cloning miR-26 sponge sequence into the minigene
Sponge26-rev	AGAggatccTTCAAGTAAAAGTGGATAGGTAGTCATTCAAGTAAAAGTGGATAGGT	
Sponge24-fwd	cgcgaattcCTGTTCCTGGACTACTGAGCCActgacCTGTTCCTGGACTACTGAGCCA	primer pair used for cloning miR-24 sponge sequence into the minigene
Sponge24-rev	AGAggatccTGGCTCAGTAGTCCAGGAACAGAGTCATGGCTCAGTAGTCCAGGAACAG	
hAGO2-sh-fwd	gatccGTCGAACATGAGACGTCATTGCTCGAGCAATGACGTCTCATGTTCGATTTTTTGg	primer pair used for cloning hAGO2 shRNA into the minigene
hAGO2-sh-rev	AATTCCAAAAAATCGAACATGAGACGTCATTGCTCGAGCAATGACGTCTCATGTTCGACG	
hAGO2-flag-fwd	cgggatccgccaccATGGACTACAAAG	primer pair used for cloning hAgo2 into the minigene
hAGO2-Not-rev	tatagcggccgcTCAAGCAAAGTACATGGTGC	

(b) Reporter gene sequences

Name	Sequence (5'-3')
GOLGA3-CA	ACCATGTTGGCCAGGCTGGTCTTGAACTCTTGACCTCAGGTGATCCTGAGCCTCAG<br>CCTCCCAAAGTGCTGGAATTATAGGCGTGAACCACCGCCCCCGGCTGCAGTTGGA<br>TTT
GOLGA3-CG	ACCATGTTGGCCAGGCTGGTCTTGAACTCTTGACCTCAGGTGATCCTGAGCCTCGG<br>CCTCCCAAAGGCTGGAATTATAGGCGTGAACCACCGCCCCCGGCTGCAGTTGGAT<br>TT
GOLGA3-GG	ACCATGTTGGCGAGGCTGGTCTTGAACTCTTGACCTCAGGTGATCCTGAGCCTCGG<br>CCTCCCAAAGGCTGGAATTATAGGCGTGAACCACCGCCCCCGGCTGCAGTTGGAT<br>TT
GOLGA3-GC	ACCATGTTGGCGAGGCTGGTCTTGAACTCTTGACCTCAGGTGATCCTGAGCCTCCG<br>CCTCCCAAAGGCTGGAATTATAGGCGTGAACCACCGCCCCCGGCTGCAGTTGGAT<br>TT
GINS1-AC	GCCTATGGCAAAACTCCGTCTCTACAAAAAATAGAAAAAATTAGCCAGGTGTGGTG<br>GTGCATGCCTGTAGTCACAGTTACACGGCAGGCTGAGGTGGGAGGATCACTTGAA<br>CCCCAGAGGTCAAGACTGCAGTGAGCTGAGATCACACCA
GINS1-GC	GCCTATGGCAAAACTCCGTCTCTACAAAAAATAGAAAAAATTAGCCAGGTGTGGTG<br>GTGCATGCCTGTAGTCACAGTTACACGGCAGGCTGGGGTGGGAGGATCACTTGAA<br>CCCCAGAGGTCAAGACTGCAGTGAGCTGAGATCACACCA
GINS1-GG	GCCTATGGCAAAACTCCGTCTCTACAAAAAATAGAAAAAATTAGCCAGGTGTGGTG<br>GTGCATGCCTGTAGTCACAGTTACACGGCAGGCTGGGGTGGGAGGATCACTTGAA<br>CCGCAGAGGTCAAGACTGCAGTGAGCTGAGATCACACCA
GINS1-CG	GCCTATGGCAAAACTCCGTCTCTACAAAAAATAGAAAAAATTAGCCAGGTGTGGTG<br>GTGCATGCCTGTAGTCACAGTTACACGGCAGGCTGCGGTGGGAGGATCACTTGAA<br>CCGCAGAGGTCAAGACTGCAGTGAGCTGAGATCACACCA
C1GALT1-A	AATGCCAACTGTATGCTAGGTACTGTGCTTAGATAATCTGTGTATAATAGTGTTAATC<br>CTCAAAATTTGGGTGTTATACCCCTATTATAGATAAGGAAACTGAGGCTCAGAGACA<br>AAGTAACTTTGTCACTGTCACAAAGCTAGTAAGTAACAGAGGTAGGAT
C1GALT1-G	AATGCCAACTGTATGCTAGGTACTGTGCTTAGATAATCTGTGTATAATAGTGTTAATC<br>CTCAAAATTTGGGTGTTGTACCCCTATTATAGATAAGGAAACTGAGGCTCAGAGACA<br>AAGTAACTTTGTCACTGTCACAAAGCTAGTAAGTAACAGAGGTAGGAT

(c) miRNA sponge sequences

Name	Sequence (5'-3')
miR-24 sponge	CTGTTCCTGGACTACTGAGCCACTGACCTGTTCCTGGACTACTGAGCCATGAC<br>TCTGTTCCTGGACTACTGAGCCA
miR-26 sponge	ACCTATCCACTTTTACTTGAACTGACACCTATCCACTTTTACTTGAATGACTACC<br>TATCCACTTTTACTTGAA
