# Supplementary material for: Structure-mediated modulation of mRNA abundance by A-to-I editing
Source: Nat Commun. 2017 Nov 2;8:1255. doi: 10.1038/s41467-017-01459-7 (PMC5665907; doi:10.1038/s41467-017-01459-7)
Supplement: Supplementary file 2 — Description of Additional Supplementary Files [file 41467_2017_1459_MOESM2_ESM.pdf]

Description of Additional Supplementary Files

File Name: Supplementary Data 1
Description: List of editing sites (included in Figure 6c), their respective gene expression levels and miRNA target information. Each editing site was required to be "edited" (editing level >0.3) in at least 3 individuals and "non-edited" (editing level = 0) in at least 3 individuals. Additionally, the closest predicted miRNA target site was required to be located within 100nt from the editing site. The relative accessibility at the miRNA target site was calculated as log2 ratio of accessibility of the "G"-containing versus "A"-containing structures, predicted using RNAplfold.

File Name: Supplementary Software 1
Description: A python script to identify the nucleotide in the opposing position of a given editing site in the RNA secondary structure predicted by RNAFold.

File Name: Supplementary Software 2
Description: A python script to calculate the relative accessibility in the RNA secondary structures containing an edited "G" nucleotide vs. a reference "A" nucleotide. This script takes the output file from Supplementary Software 1 as an input file.
